# Radioecological and geochemical peculiarities of cryoconite on Novaya Zemlya glaciers

**DOI:** 10.1038/s41598-021-02601-8

**Published:** 2021-11-29

**Authors:** Alexey Miroshnikov, Mikhail Flint, Enver Asadulin, Ramiz Aliev, Andrei Shiryaev, Arsenii Kudikov, Vladimir Khvostikov

**Affiliations:** 1 Institute of Geology of Ore Deposits, Petrography, Mineralogy, and Geochemistry RAS, Moscow, 119017 Russia; 2 Shirshov Institute of Oceanology RAS, Moscow, 117997 Russia; 3 National Research Center “Kurchatov Institute”, Moscow, 123182 Russia; 4 Chemistry Department, Lomonosov Moscow State University, Leninskie Gory, 1, Moscow, 119991 Russia; 5 Frumkin Institute of Physical Chemistry and Electrochemistry RAS, Moscow, 119071 Russia; 6 Institute of Geography RAS, Moscow, 119017 Russia; 7 Institute of Microelectronics Technology Problems and High-Purity Materials RAS, Chernogolovka, Moscow Region 142432 Russia

**Keywords:** Ecology, Environmental sciences

## Abstract

In recent years, cryoconite has received growing attention from a radioecological point of view, since several studies have shown that this material is extremely efficient in accumulating natural and anthropogenic radionuclides. The Novaya Zemlya Archipelago (Russian Arctic) hosts the second largest glacial system in the Arctic. From 1957 to 1962, numerous atmospheric nuclear explosions were conducted at Novaya Zemlya, but to date, very little is known about the radioecology of its ice cap. Analysis of radionuclides and other chemical elements in cryoconite holes on Nalli Glacier reveals the presence of two main zones at different altitudes that present different radiological features. The first zone is 130–210 m above sea level (a.s.l.), has low radioactivity, high concentrations of lithophile elements and a chalcophile content close to that of upper continental crust clarkes. The second zone (220–370 m a.s.l.) is characterized by high activity levels of radionuclides and “inversion” of geochemical behaviour with lower concentrations of lithophiles and higher chalcophiles. In the upper part of this zone (350–370 m a.s.l.), ^137^Cs activity reaches the record levels for Arctic cryoconite (5700–8100 Bq/kg). High levels of Sn, Sb, Bi and Ag, significantly exceeding those of upper continental crust clarkes, also appear here. We suggest that a buried layer of contaminated ice that formed during atmospheric nuclear tests serves as a local secondary source of radionuclide contamination. Its melting is responsible for the formation of this zone.

## Introduction

Cryoconite is a promising environmental matrix to study the radioecology of the Arctic and other glacial regions^1,2^. Sediments in the bottoms of cryoconite holes usually consist of dark, often black granules, which are minerals (80–98 mass%) and organic matter (2–20 mass%)^3^. Cryoconite absorbs solar radiation, and associated melting leads to the formation of cylindrical holes in the glacier surface. The ability of cryoconite to accumulate natural and anthropogenic radionuclides from the atmosphere to very high activity levels has been shown in a number of publications^1,2,4–14^.

**Figure 1:**
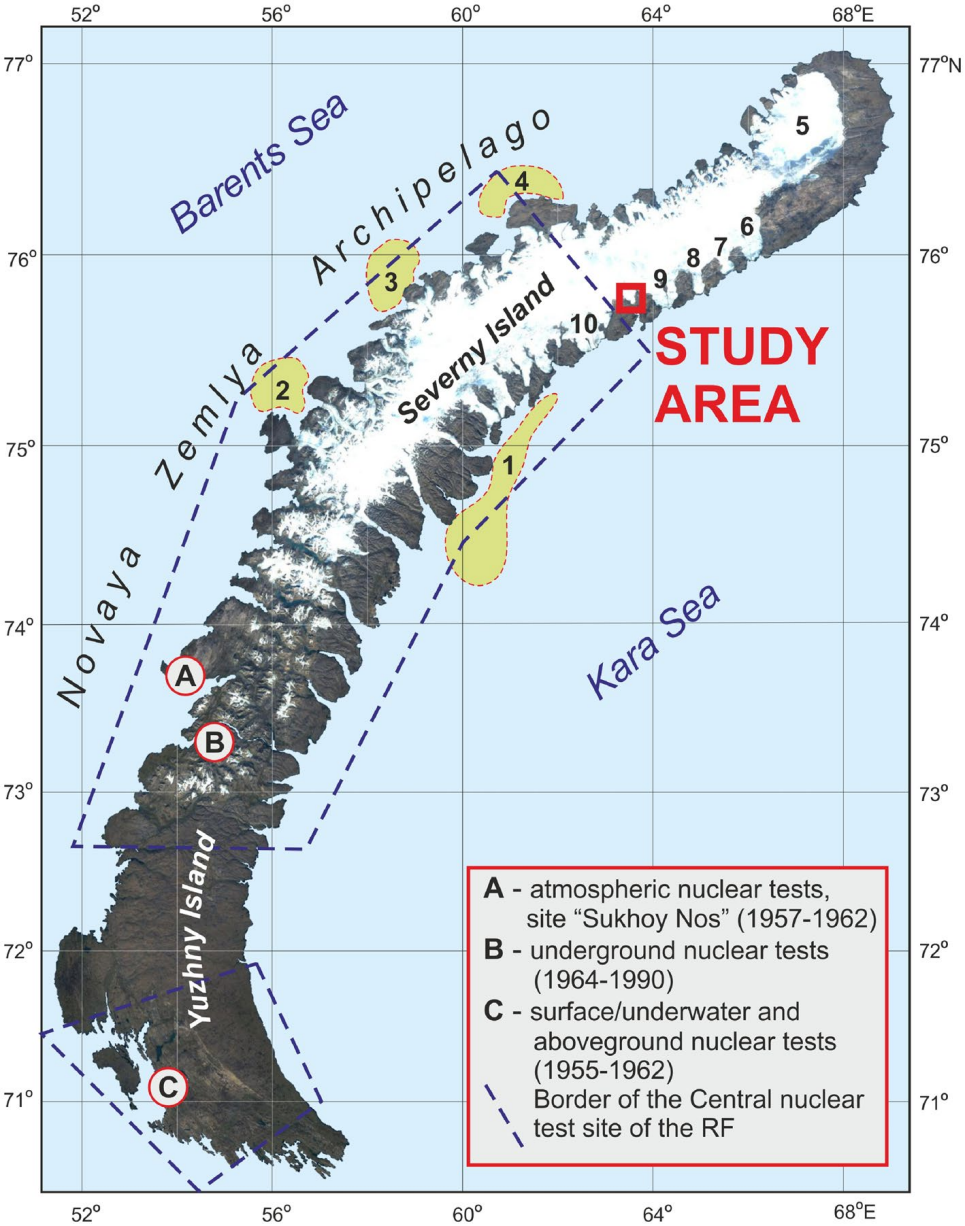
Location of the sampling site on Nalli Glacier (red box). Zones of high ^137^Cs activity in bottom sediments: 1—by Miroshnikov^23^; 2—by Crane et al.^24^; 3 and 4—by Ivanov^25^; glaciers: 5—northern icecap; 6—Roze, 7—Sredny, 8—Rozhdestvensky, 9—Vershinsky, and 10—Moshchny. The cartographic base was obtained from Sentinel-2 cloudless (https://s2maps.eu) using EOX IT Services GmbH (Contains modified Copernicus Sentinel data from 2016 to 2017). The figure was created using Corel Draw X7 software (https://www.coreldraw.com).

The modern glaciated area at Novaya Zemlya is the second largest in the Northern Hemisphere after the Greenland ice sheet in terms of volume and area, and covers ~ 22,000 km^2,15,16^. A significant share of both islands of the archipelago belongs to the Central Test Site of the Ministry of Defence of the Russian Federation (Fig. 1). In total, 130 nuclear tests were performed in 1957–1990. In particular, on the “Sukhoy Nos” test ground (A in Fig. 1), 88 atmospheric tests with a total yield of > 250 Mt were performed in 1957–1962. Various types of underground, underwater and atmospheric tests occurred at sites B and C (Fig. 1)^17,18^. The explosions generally included various environmental safety measures. In particular, meteorological conditions were selected to limit the extent of radioactive fallout to the test site, and the major fraction was confined to the Severny Island^17,19^.

After a nuclear explosion, fallout of fission products, including ^137^Cs, occurs in three stages: large particles are removed from the troposphere within hours, the residence time of small particles in the troposphere ranges from a few weeks to several months, and the residence time of aerosols in the stratosphere can reach 2–3 years or more^20^. Small amounts of radioactive products can be detected in atmospheric fallout even today, but their presence is apparently associated with resuspension from the surface. The tropospheric component falls to the Earth’s surface locally or within a local region, while the stratospheric component is distributed across the hemisphere and determines the density of the global fallout of anthropogenic radionuclides, which peaked in 1963^21^. The large size of the Severny Island and variations in meteorological and test parameters lead to heterogeneities in the density of fallout radioactivity. The fraction that falls on the surfaces of glaciers outside their accumulation zones and on surrounding soil is mobilized by meltwater and migrates towards the Kara and Barents seas^22^. This hypothesis is based on our study of 1995–2003 Kara Sea radiation conditions, which identified a high concentration zone of radiocesium in bottom sediments sampled near the southeastern coast of Severny Island^23^ (1 in Fig. 1). Subsequent works^24,25^ provided solid support for this scenario: similar zones of high ^137^Cs activity were found on the other side of the island, in the bottom sediments of the Barents Sea (2, 3, and 4 in Fig. 1).

A fallout fraction, which fell into the accumulation zone, was firmly tied to seasonal precipitation and participated in the formation of contaminated ice layers. Presumably, such a layer that formed in the course of nuclear tests is still present in Novaya Zemlya glaciers and is a potential rich reservoir of anthropogenic radioactivity. Our attempt to detect this layer in ice cores drilled in 2015 at the North Glacier Dome (5 in Fig. 1) was unsuccessful. However, these glaciological studies allowed us to estimate the depths of the radiation-contaminated layer in this part of the glacier as 25–30 m in the cold firn zone and ~ 15–20 m at the boundary of the accumulation zone^26^. Taking into account that cryoconite holes are abundant in the ablation region of glaciers and are known as efficient accumulators of natural and anthropogenic radionuclides, we initiated a detailed investigation of cryoconite in Nalli Glacier (red box in Fig. 1, Supplementary Fig. S1). This study aims to explore the relationship between the notable radioactive contamination that occurred in Novaya Zemlya and the concentration of radionuclides in cryoconite collected from its glaciers. The primary assumption on which this study is based is that cryoconite is extremely efficient in accumulating radionuclides from meltwater, as highlighted by previous publications^1,2,6,9^. This work presents the first results of a study of anthropogenic (^137^Cs, ^241^Am, and ^207^Bi) and natural (^210^Pb and ^7^Be) radionuclides trapped in cryoconite on the Nalli Glacier and their geochemical features, which were studied for 50 macro- and microelements. This is the first such study of cryoconite of the Russian Arctic sector.

## Results and discussion

**Figure 2:**
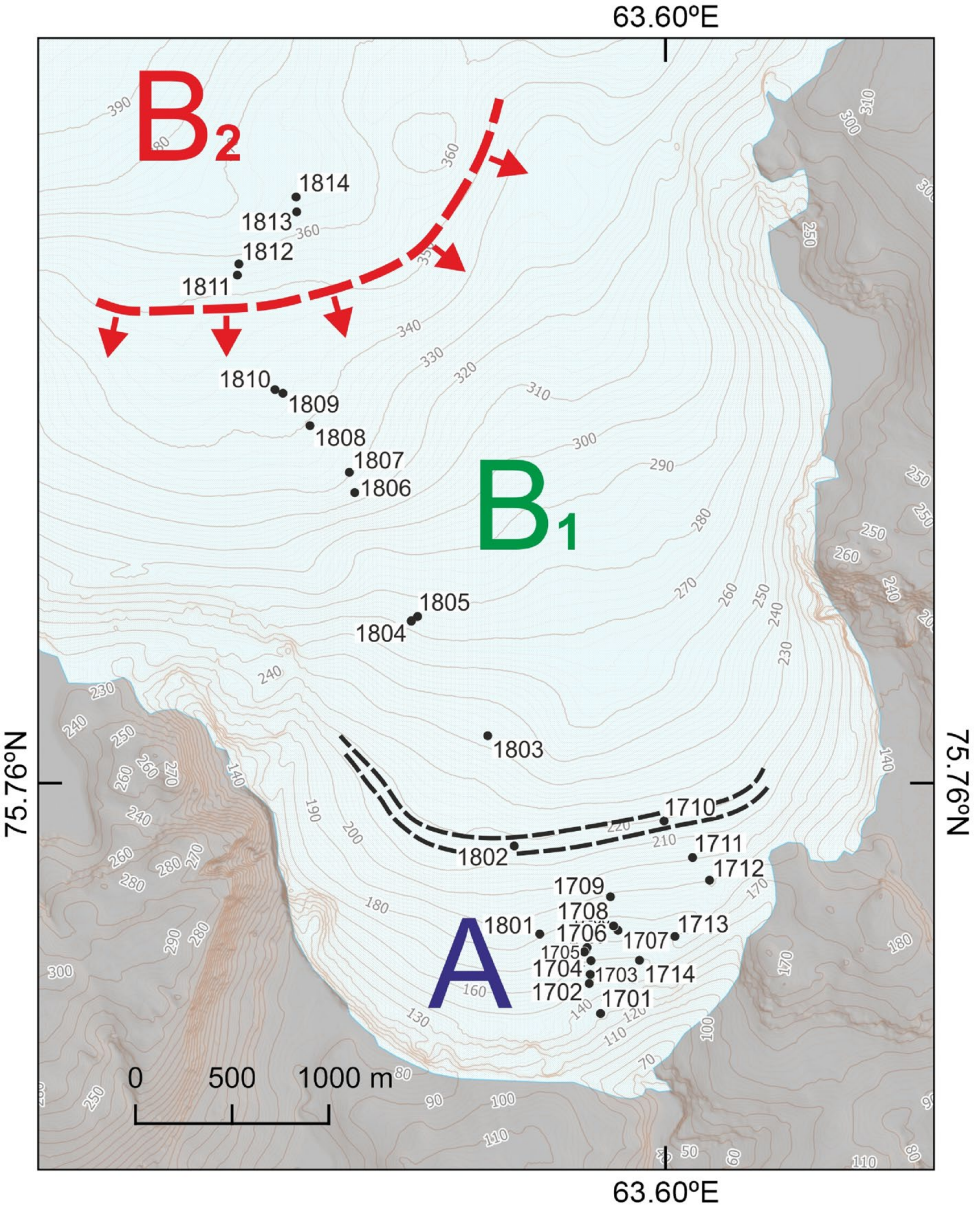
Location of sampling points on Nalli Glacier. A—^137^Cs activity zone < 440 Bq kg^−1^; B_1_—^137^Cs activity zone 2700–4660 Bq kg^−1^; B_2_—^137^Cs activity zone 5700–8100 Bq kg^−1^. The black dashed lines indicate the narrow transitional area between zones A and B. The red dashed line with arrows represents the frontal part of secondary radioactive contamination (see text for details). 1701–1714—samples collected in 2017; 1801–1814—samples collected in 2018. The relief and contours were obtained from ArcticDEM. DEM(s) were created from DigitalGlobe, Inc., imagery and funded under National Science Foundation awards 1043681, 1559691, and 1542736. The elevation map was created using QGIS 3.16 (https://www.qgis.org). The figure was created using Corel Draw X7 software (https://www.coreldraw.com).

**Figure 3:**
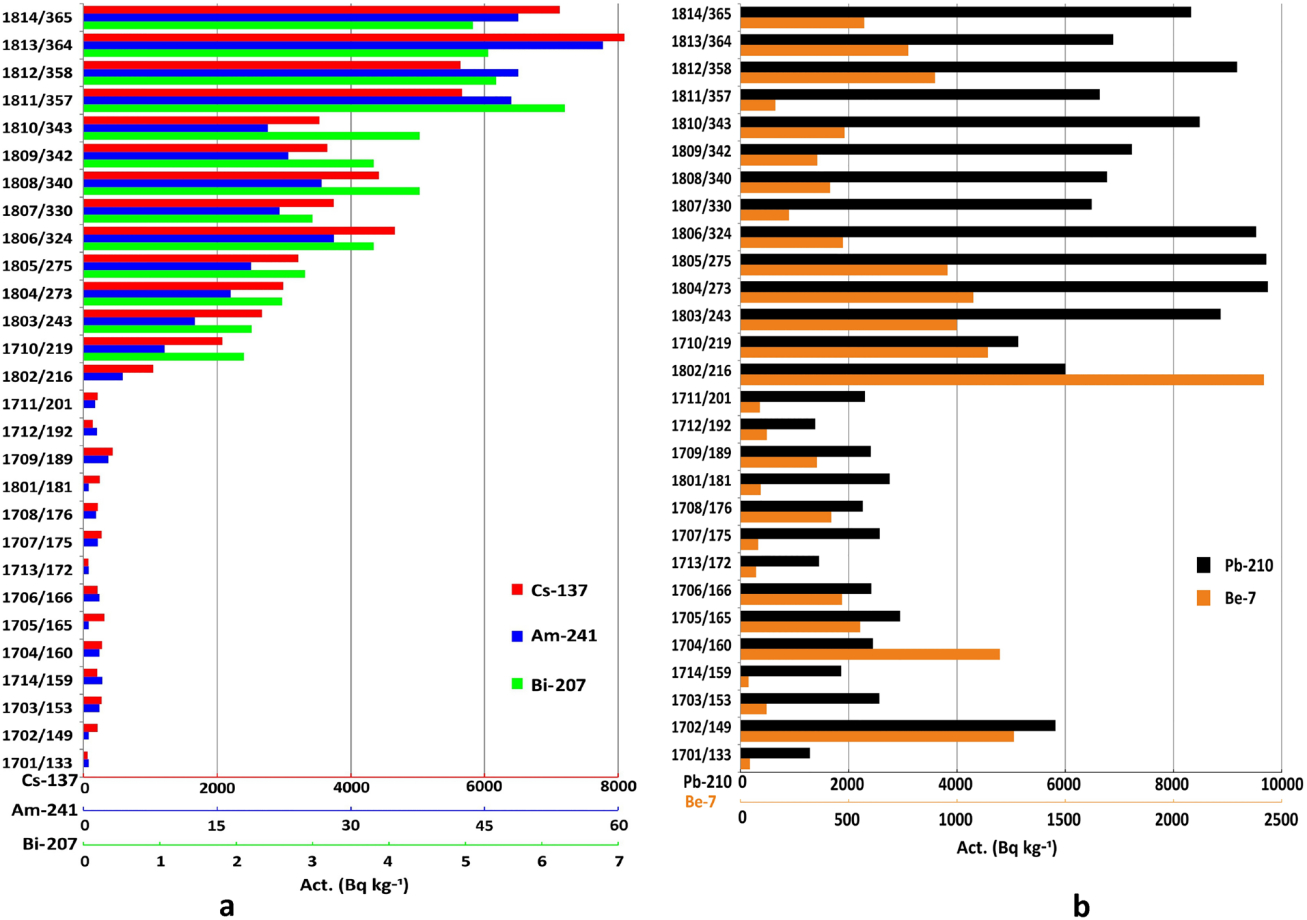
Activity of anthropogenic (^137^Cs: red, ^241^Am: blue, and ^207^Bi: green) (**a**) and natural (^210^Pb: black and ^7^Be: orange) (**b**) radionuclides in cryoconite samples. Horizontal axis—activity of radionuclides (Bq kg^−1^); vertical axis—sample/altitude (m a.s.l.).

Data for all analysed radionuclides are presented in the “Supplementary Material”. Cryoconite samples were collected on Nalli Glacier (Supplementary Fig. S1) on Sept. 25, 2017 (samples 1701–1714) and on Sept. 10, 2018 (samples 1801–1814) at 28 spots (Fig. 2, Supplementary Table S1). Gamma spectrometric analysis of samples showed the presence of anthropogenic radionuclides ^137^Cs, ^241^Am, and ^207^Bi. All quoted radioactivity values were recalculated for the sampling date, except those for ^241^Am since the concentration of the parent ^241^Pu isotope is unknown. However, for this isotope, the correction for decay is negligible. The activity of ^137^Cs reached 8093 (± 69) Bq kg^−1^ of dry weight, that of ^241^Am reached 58.3 (± 2.3) Bq kg^−1^ and that of ^207^Bi reached 6.3 (± 0.6) Bq kg^−1^. The natural radionuclides ^210^Pb and ^7^Be were also present in all samples. The activity of ^210^Pb varied in the range of 1280–9750 Bq kg^−1^. In addition, in the investigated samples, a significant amount of short-lived cosmogenic radionuclide ^7^Be was found, whose specific activity reached 2418 (± 76) Bq kg^−1^ (Fig. 3, Supplementary Table S2). To evaluate the contribution of atmospheric components to the total ^210^Pb activity, ^226^Ra activity was determined and found to be 17–27 Bq kg^−1^ (Supplementary Table S2). Based on the ^210^Pb/^226^Ra ratio, we conclude that more than 98% of ^210^Pb was of atmospheric provenance.

### Anthropogenic radionuclides

A specific radioactivity of ^137^Cs exceeding 8000 Bq kg^−1^ is the record for Arctic cryoconite. It is known that levels of ^137^Cs activity in cryoconite can reach even more extreme values of 140 kBq^6^ and even 223 kBq^13^. However, the data in Wilflinger et al.^13^ were recalculated for 01.05.1986, and those in Tieber et al*.*^6^ were recalculated for 03.10.2006. At present, both values would be approximately 100 kBq each. In addition, it should be noted that these data were obtained from Schladming Glacier and Stubacher Sonnblick Glacier, which are located in a relatively restricted area in the European Alps that had the highest density (reaching 1480 kBq m^−2^) of radioactive fallout from the Chernobyl accident in 1986^27^. Both glaciers (47° 28′ N and 47° 07′ N) are located in the latitudinal belt with the highest density of global fallout in the Northern Hemisphere, exceeding this parameter by ~ 13 times compared to Arctic latitudes 70–80° N at which the Nalli Glacier is situated^28^. Accordingly, based on generally accepted latitudinal zonation, we can assume that with the exception of spots directly affected by radiation accidents and/or nuclear tests^20^, the ^137^Cs activity levels in cryoconite from Arctic glaciers should generally reflect the global fallout level. This hypothesis is supported by literature data and eleven additional samples collected by us on Svalbard glaciers Jotufonna, Vestre Grønfjordbreen and Fridtjovbreen in the framework of another project (Table 1). Cryoconite samples from Svalbard glaciers were added to this work to better interpret our results (Supplementary Table S3).

**Table 1:** Maximum and mean levels of ^137^Cs radioactivity (Bq kg^−1^) in Arctic glacier cryoconite.

Glacier/sampling year	Region	Latitude (N)	Max act. ^137^Cs	Mean act. ^137^Cs	Data source	(n)
Waldemar Glacier/2014	Svalbard	78° 40ʹ	2030 (± 257)	721 (± 109)	Łokas et al.^2^	12
Jotufonna Glacier/2017	Svalbard	78° 37ʹ	1114 (± 10)	459 (± 3)	This study (Table S3)	5
Vestre Grønfjord Glacier/2018	Svalbard	77° 56ʹ	111 (± 2)	108 (± 2)	This study (Table S3)	2
Fridtjov Glacier/2018	Svalbard	77° 49ʹ	1781 (± 16)	610 (± 6)	This study (Table S3)	4
Scott Glasier/2005	Svalbard	77° 33ʹ	285 (± N/A)	285 (± N/A)	Chmiel et al.^29^	1
Werenskiold Glacier/2016	Svalbard	77° 04ʹ	4500 (± 20)	1700 (± 60)	Łokas et al.^11^	5
Hans Glacier/2014	Svalbard	77° 01ʹ	678 (± 91)	356 (± 58)	Łokas et al.^10^	15
Nalli Glacier/2017	Novaya Zemlya	75° 46ʹ	2076 (± 69)	354(± 4)	This study (Table S2)	14
Nalli Glacier/2018	Novaya Zemlya	75° 46ʹ	8093 (± 69)	4049(± 33)	This study (Table S2)	14

Three other studies of ^137^Cs in Northern Hemisphere cryoconite may be mentioned. On Russell Glacier in Greenland (67° 09′ N) the maximum ^137^Cs activity was 123 (± 93) Bq kg^−1^^30^, on Castle Creek Glacier located 2670 km south of ‘our’ latitude belt (53° 02′ N) in Canada—3969 (± 149) Bq kg^−1^^31^, and on Isfallsglaciären (67°, 54′ N) in Arctic Sweden—4533 (± 149) Bq kg^−1^^14^. A comparison of our results with radiocesium activity data given in Table 1 shows that the determined maximum activity level of ^137^Cs of 8100 Bq kg^−1^ in sample 1813, as well as at three nearby locations from 5665 to 7125 Bq kg^−1^, is currently the highest recorded for Arctic glaciers. The highest activity detected in one sample for Svalbard cryoconite on Werenskiold Glacier (no exact coordinates are given) is 4500 Bq kg^−1^^11^. Taking this value as the maximum for the global deposition level, and considering the difference in sampling time, it is two times lower than that at Nalli Glacier. Accordingly, at least four samples (1811, 1812, 1813, and 1814) of Nalli Glacier cryoconite (Supplementary Table S2) contain not only a global fallout contribution but also an additional contribution, which, according to our assumptions, is now being released from the contaminated layer that formed due to local fallout in the accumulation zone from nuclear tests.

**Figure 4:**
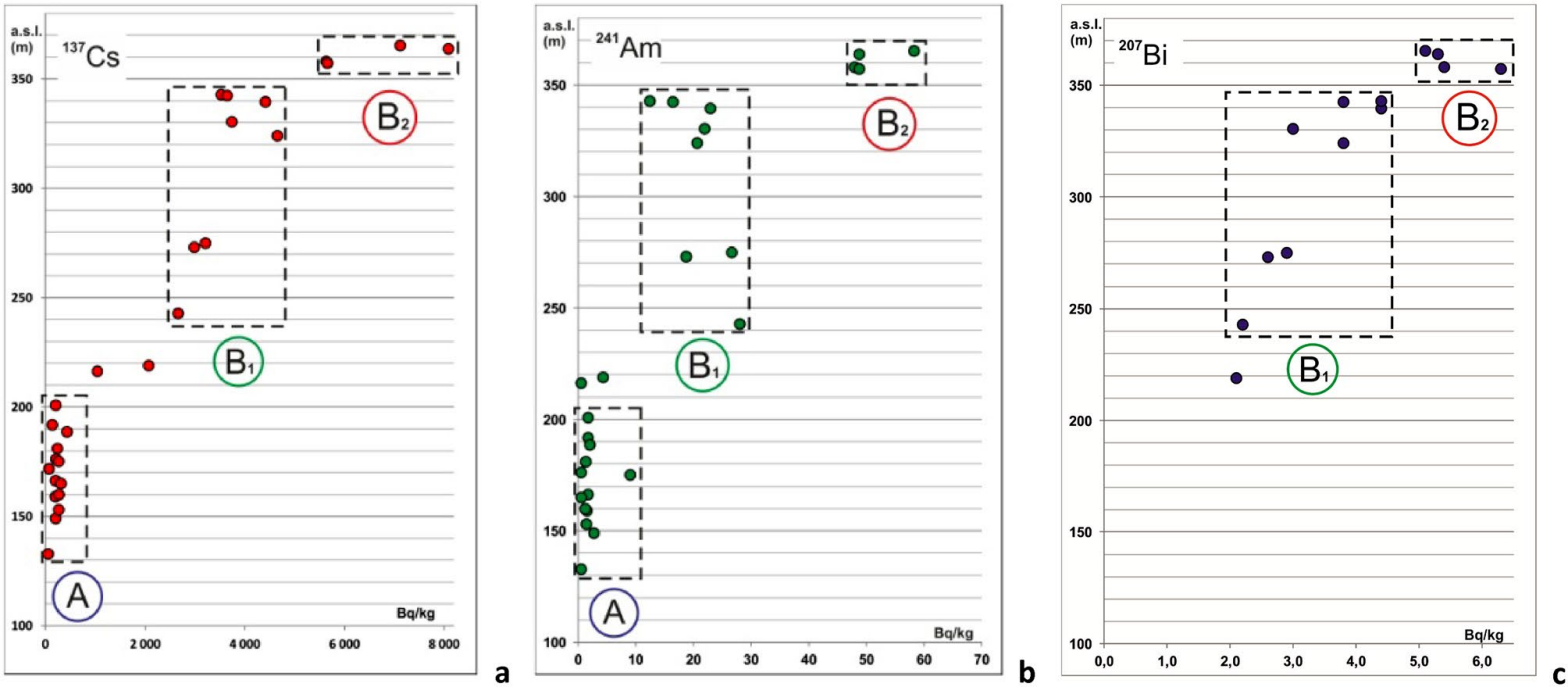
Distribution of the specific activity of anthropogenic radionuclides in cryoconite as a function of altitude on the surface of the Nalli Glacier: (**a**)—A_Cs-137_ (m a.s.l.); (**b**)—A_Am-241_ (m a.s.l.); (**c**)—A_Bi-207_ (m a.s.l.).

Analysis of the distribution of ^137^Cs, ^241^Am and ^207^Bi activities in cryoconite relative to the hypsometric levels of point locations on the glacier surface leads to their division into three groups, termed zones A, B_1_, and B_2_ (Fig. 4). The first group of cryoconite holes consists of densely clustered points up to 210 m a.s.l., where ^137^Cs activity varies from 58 to 436 Bq kg^−1^ and ^241^Am from 1.3 to 9.1 Bq kg^−1^ (A in Fig. 4a/b). No ^207^Bi is detected. Cryoconite holes located between 240 and 345 m a.s.l. (B_1_ in Fig. 4) compose the second group. In this group, the radioactivity of ^137^Cs ranges from 2667 to 4659 Bq kg^−1^, and ^241^Am is in the range of 12.5–28.1 Bq kg^−1^ (B_1_ in Fig. 4a/b); these values are quite consistent with data from the Waldemar Glacier^2^. In this group, ^207^Bi appears with specific activities ranging from 2.2 to 4.4 Bq kg^−1^ (B_1_ in Fig. 4c). It should be noted that only a few works address ^207^Bi in the natural environment, and this study is the first to show its presence in Arctic cryoconite. The origin of this radionuclide is still the subject of debate. Noshkin et al*.*^32^ showed the absence of a direct link between the yield of an explosive device and the amount of ^207^Bi released. Based on soil data in the Faroe Islands Aarkrog et al*.* suggested that a high-power thermonuclear bomb at the Novaya Zemlya test site on October 30, 1961, and other so-called “clean” thermonuclear weapons are the most likely sources of ^207^Bi^33^.

Two samples 1710 and 1802 located at altitudes of 210–220 m a.s.l., form an intermediate zone between the groups mentioned above. On the glacier itself, this area is characterized by pronounced ridges and grooves with directions normal to the glacier flow direction; additionally the surface slope changes: in zone A the surface is 3°–3.5° steeper. The environment containing the cryoconite holes in zone A is more dynamic with larger amounts of meltwater and more intense flows. In the lower part of the glacier, the holes are less stable and contain smaller amounts of organics. Consequently, zone A is less suitable for the accumulation of radionuclides. A third group of cryoconite holes is located above 350 m a.s.l. (B_2_ in Fig. 4). Here, ^137^Cs activity reaches 8100 Bq kg^−1^ (B_2_ in Fig. 4a), ^241^Am—58 Bq kg^−1^ (B_2_ in Fig. 4b), and ^207^Bi—6.3 Bq kg^−1^ (B_2_ in Fig. 4c). Apparently, the cryoconite samples in this group are enriched with “additional” radionuclides ^137^Cs, ^241^Am and ^207^Bi relative to the B_1_ zone and are the leading frontal part of the radioactive material released from the radiation-contaminated layer.

**Figure 5:**
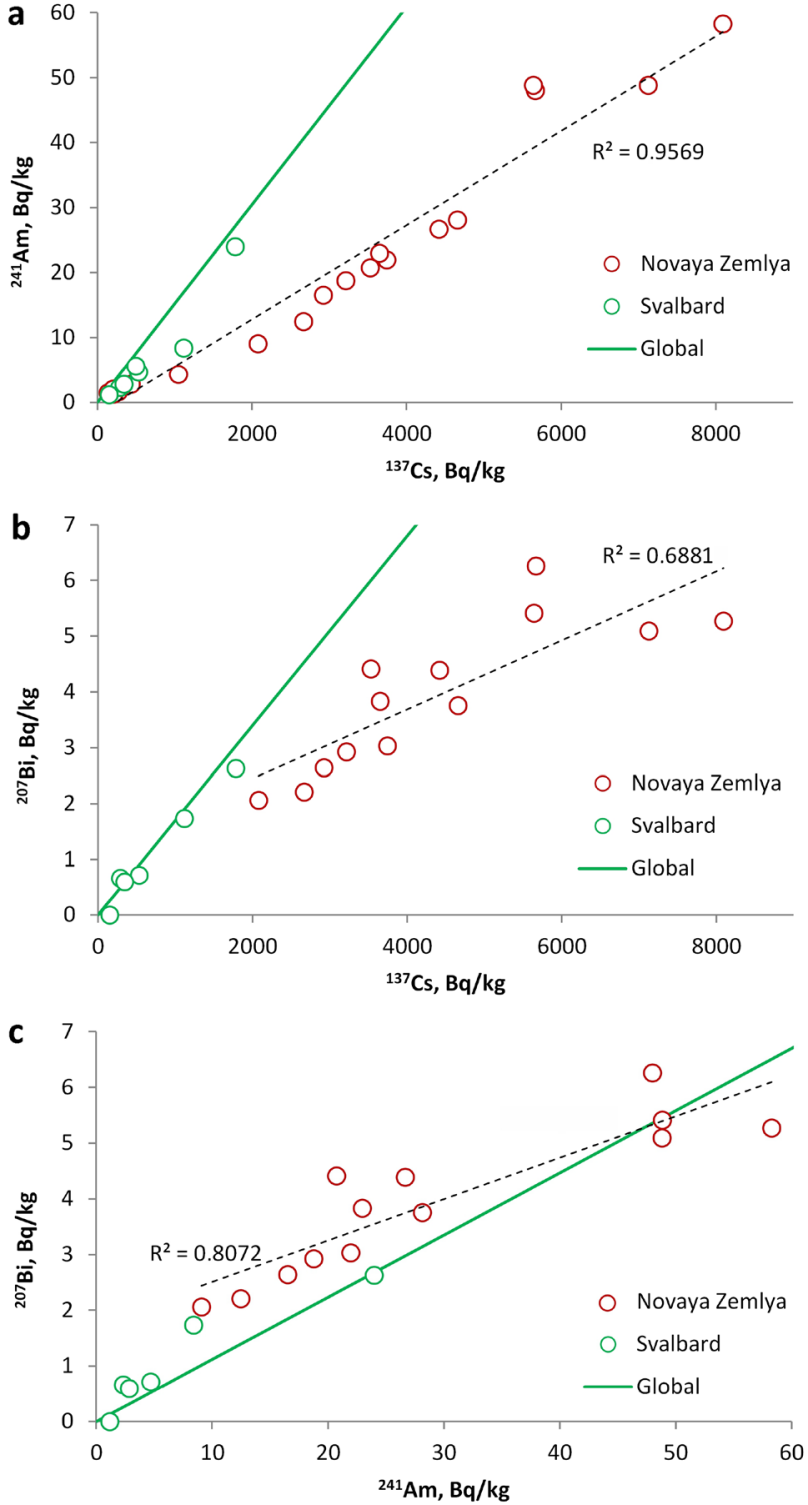
Radionuclides in cryoconite samples from Novaya Zemlya and Svalbard. (**a**)—^241^Am/^137^Cs ratio; (**b**)—^207^Bi/^137^Cs; (**c**)—^207^Bi/^241^Am. Black dashed line—corresponding trends. The solid black line corresponds to global fallout^38–41^.

The zonal distribution of cryoconite into three groups becomes more prominent when considering the ^241^Am/^137^Cs (Fig. 5a), ^207^Bi/^137^Cs (Fig. 5b) and, especially, ^207^Bi/^241^Am (Fig. 5c) ratios. The ^241^Am/^137^Cs ratio (Fig. 5a) is used to distinguish Chernobyl fallout from global levels^34^. In the Chernobyl accident relatively small amounts of transuranic elements were released into the atmosphere, mostly in the form of relatively large 'hot' particles that fell in a relatively small area around the reactor^35^. A significant fraction of volatile radionuclides, including ^137^Cs, were released in aerosol form and distributed worldwide^36,37^. ^241^Am is not produced directly in nuclear explosions; instead, it results from ^241^Pu decay. There are few data on the presence of the latter in natural objects because its measurement is complicated. The activity of ^241^Pu delivered to the environment is more difficult to estimate than that of fission fragments since its production in any given explosion depends both on the explosion yield and on the initial isotopic composition of the charge. It has been estimated^34^ that atmospheric nuclear explosions have released 142 PBq of ^241^Pu into the environment. Most of it fell out between 1961 and 1964, and by now, it has almost all decayed. The decay of ^241^Pu leads to an increase in ^241^Am activity, which will pass through a maximum in 2036^34^. Both ^241^Am and ^241^Pu are firmly bound to suspended matter, so their fractionation after fallout is unlikely.

A recent paper^38^ gives a global fallout ratio of ^241^Am/^239+240^Pu = 0.37, as recalculated for 2018. The ^137^Cs/^239+240^Pu ratio was determined in several papers^39,40^. Recalculated for 2018, it is ≈ 24. Based on these sources, we estimate that the global activity ratio ^137^Cs/^241^Am ≈ 0.015. Analysis of 26 samples of cryoconite and alpine soils gave the following ratio ^207^Bi/^137^Cs_(global)_ = (1.70 ± 0.12) × 10^–3^^41^. In the five samples from Svalbard (our unpublished data), the average ^207^Bi/^137^Cs ratio was (1.7 ± 0.4) × 10^–3^, which correlates well with^41^ (Fig. 5b). In the cryoconite of Novaya Zemlya, however, this ratio is noticeably lower, (9.2 ± 1.6) × 10^–4^ (n = 13), which is closer to the ratio given in Ref.^33^. For the Stubacher and Hallstatter alpine glaciers, the values of ^207^Bi/^137^Cs_(global)_ are (1.00 ± 0.37) × 10^–3^ and (2.75 ± 1.24) × 10^–3^, respectively^13^. Note that the ratio of ^207^Bi/^137^Cs in natural objects may not correspond to the original composition of the fallout but may result from secondary fractionation processes^41^. This may explain some of the variation in the values obtained in different regions. It is natural to assume that fractionation should increase the ^207^Bi/^137^Cs ratio since ^137^Cs is a more mobile radionuclide than ^207^Bi. According to Aarkrog^33^, the super high-power explosion on Novaya Zemlya was the main global source of ^207^Bi. This assumption is indirectly supported by the higher activity of ^207^Bi in Novaya Zemlya than in Svalbard. However, our results do not permit us to make firm conclusions about the origin of this nuclide. Variations in the ^207^Bi/^137^Cs ratio in different regions may result both from the existence of several sources (e.g., high-yield thermonuclear explosions) and from the markedly different geochemical behaviour of these elements. More detailed studies are required to unambiguously identify the origin of artificial radionuclides on Novaya Zemlya involving other matrices such as peat, soils, lichens, and mosses.

### Natural radionuclides

**Figure 6:**
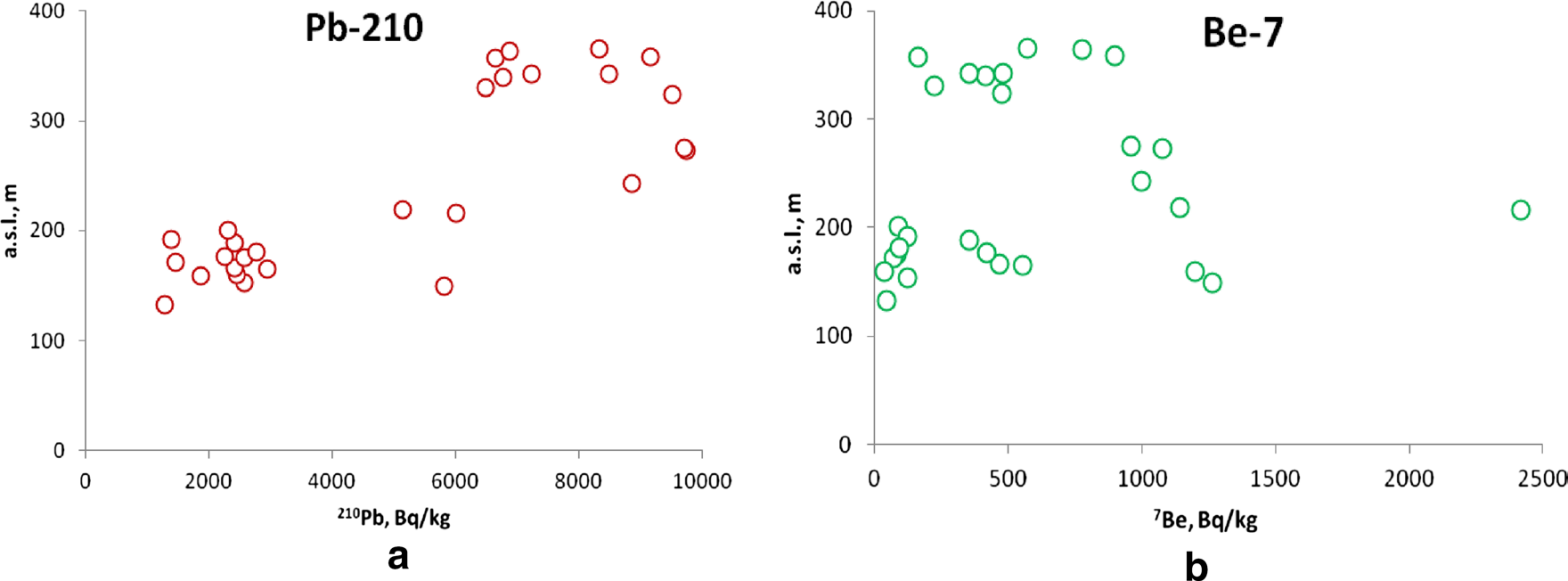
Distribution of specific activity of ^210^Pb (**a**) and ^7^Be (**b**) in Nalli Glacier cryoconite depending on altitude above sea level.

Cryoconite holes on a glacier surface may survive for years^42^, accumulating precipitated matter, radionuclides of various origins in particular, which are transported into cryoconite via meltwater. For example, high concentrations of ^210^Pb (T_1/2_ = 22.2 years) and ^7^Be (T_1/2_ = 53.2 days) have been reported in cryoconite^1,2,8–12,14^. ^210^Pb results from Rn decay, which, in turn, is produced from Ra present in rocks and soils in trace amounts. Cosmogenic ^7^Be is produced via the interaction of cosmic rays with the upper troposphere and stratosphere. Both nuclides tend to fix in particulate matter, but markedly different half-lives permit the use of them to estimate the residence time of cryoconite holes^13^. Almost all our samples contain both isotopes. In contrast to other radionuclides, the spatial distribution of ^7^Be does not show marked peculiarities or zoning (Fig. 6b). Its activity is in the range of 38 (± 10)–1264 (± 45) Bq kg^−1^, with the exception of sample 1802, which contains 2418 (± 76) Bq kg^−1^. The old-inactive cryoconite, having no contact with meltwater, is devoid of ^7^Be^9^. The presence of ^7^Be implies that on the date of sampling the cryoconite was “active”, i.e., was accumulating radionuclides. Consequently, cryoconite holes in Nalli Glacier accumulate radionuclides independently of their location on the glacier. This highlights that the supra-glacial environment of Nalli Glacier is dynamic from a hydrologic point of view, with a network of supraglacial channels capable of spreading radionuclides in cryoconite across the entire glacier.

In contrast, ^210^Pb behaves differently. The specific activity of this nuclide varies in the range of 1283 (± 31)–9748 (± 274) Bq kg^−1^ (Supplementary Table S2). Similar to anthropogenic radionuclides, it clearly correlates with hypsometric position: spots with lower activity (1200–3000 Bq kg^−1^) mostly lie below 210 m a.s.l. (Fig. 6a). This behaviour likely indicates that cryoconite in the upper part of the glacier is older than in the lower part, possibly due to removal of the latter with meltwater and rain. The lower part of the glacier is, in general, more dynamic than the upper part. Cryoconite holes above 220 m a.s.l. appear to be more stable and survive several seasons, accumulating significant amounts of ^210^Pb.

### Phase composition and selected properties of cryoconite samples

**Figure 7:**
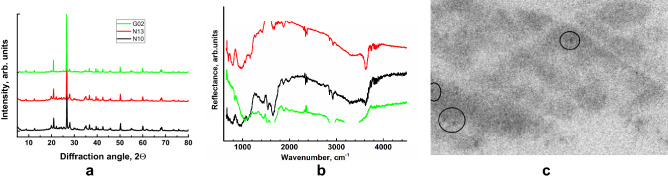
Representative X-ray diffraction patterns (**a**) and infrared spectra (**b**) for cryoconite from Novaya Zemlya (N10 and N13 correspond to 1710 and 1713—Nalli Glacier, zone A) and Svalbard (G02—Vestre Grønfjord Glacier). The curves are displaced vertically for clarity. Autoradiograph (**c**) of a powdered cryoconite sample (horizontal size 5 cm). The darkening is uniform and precisely matches the distribution of the powder. Circles mark spots with higher activity.

The X-ray diffraction patterns of the three studied samples are virtually identical (Fig. 7a). Semiquantitative phase analysis gives 40% quartz and 17% albite, and the rest is represented by micas (chlorite, muscovite, biotite, and phlogopite). These results are consistent with data on cryoconites from other polar regions^43,44^. Note that the sample preparation complicates the quantitative analysis of micas due to possible texturing.

Infrared spectra (Fig. 7b) are dominated by micas (e.g., phlogopite): bands are due to Si–O and Si–O–Si (Al, Mg) between 690 and 1200 cm^−1^^45^, Si–O stretch overtones are within 1650–1870 cm^−1^, and aliphatic C–H bonds (2850 and 2920 cm^−1^) and OH groups (2900–3650 cm^−1^) are present. In contrast to the XRD results, the samples demonstrate considerable scatter in the abundance of various species. This clearly results from a much smaller beam size (here—100 µm) than in the XRD case (approx. 1 cm^2^). Since cryoconites consist of a mixture of minerals and biological films, the results of micro-IR spectroscopy are very sensitive to the selection of the studied grains. To obtain sample-averaged information, preparation of KBr-based pellets is required. However, in our view, microspectroscopy is an extremely valuable tool for cryoconite research, since after proper sample preparation, it allows examination of different types of granules (e.g., those suggested by Takeuchi et al*.*^46^) and interaction of organic matter with individual minerals.

An important supplement to the precise study of radionuclides was the investigation of spatial distribution of radioactivity in cryoconite samples using digital autoradiography. Autoradiography of a large set of samples from various locations (Novaya Zemlya and Svalbard) gave similar results—a rather uniform distribution of weak levels of activity. However, in some samples, “hot spots” with activity approximately two times higher than that of the rest of the sample were observed (Fig. 7c). The splitting method was applied to extract some “hot particles” for subsequent gamma spectroscopy, but the activity of separated mineral grains was extremely low, ruling out the presence of radioactive particles from historic nuclear explosions. Some information about the origin of these “hot spots” was provided by comparison of autoradiographs with µ-XRF mapping. In the studied cryoconite samples, the “hot spots” are associated either with small (10–20 µm) Zr-rich grains (likely zircon) or with Fe-containing particles, most likely Fe oxides. Zircon often contains an admixture of uranium and/or thorium, and thus observation using autoradiography is expected. Explanation of the radioactivity of Fe-containing grains is less certain, but sorption of Pu and some other radionuclides on Fe oxides may be important in a wide range of environmental conditions^47^.

### Geochemical characteristics of cryoconite

**Figure 8:**
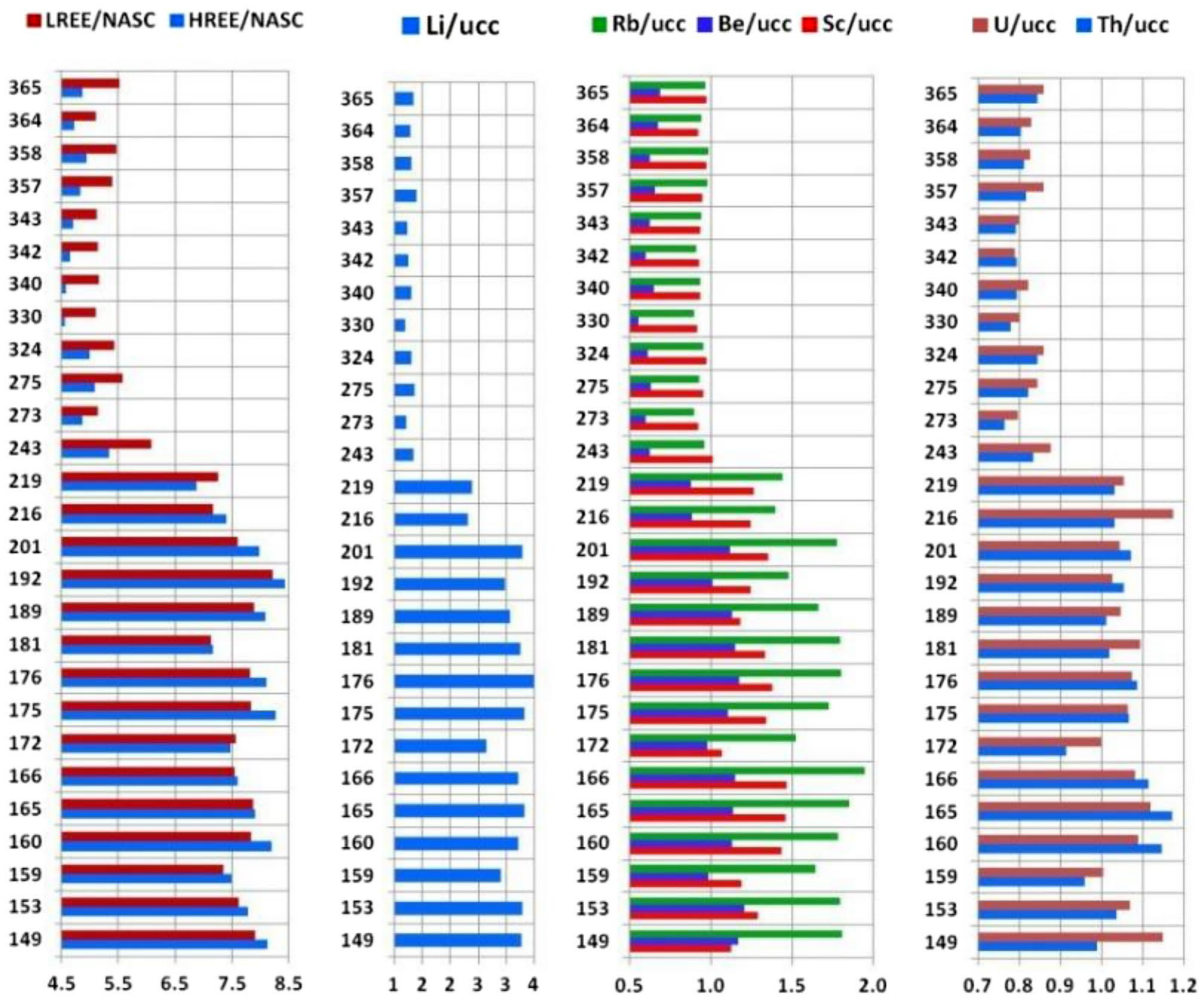
Distribution of lithophile element contents in Nalli Glacier cryoconite samples from altitudes of 149–365 m a.s.l. LREE = ∑(La, Ce, Pr, Nd, Sm, Eu, Gd); HREE = ∑(Tb, Dy, Ho, Er, Tm, Yb, Lu). The vertical axis shows the absolute altitude of the sampling spot, and the horizontal axis shows the normalized contents of the elements. Normalization standards: NASC—North American Shale Composite^50^, UCC—Upper Continental Crust^48^.

**Figure 9:**
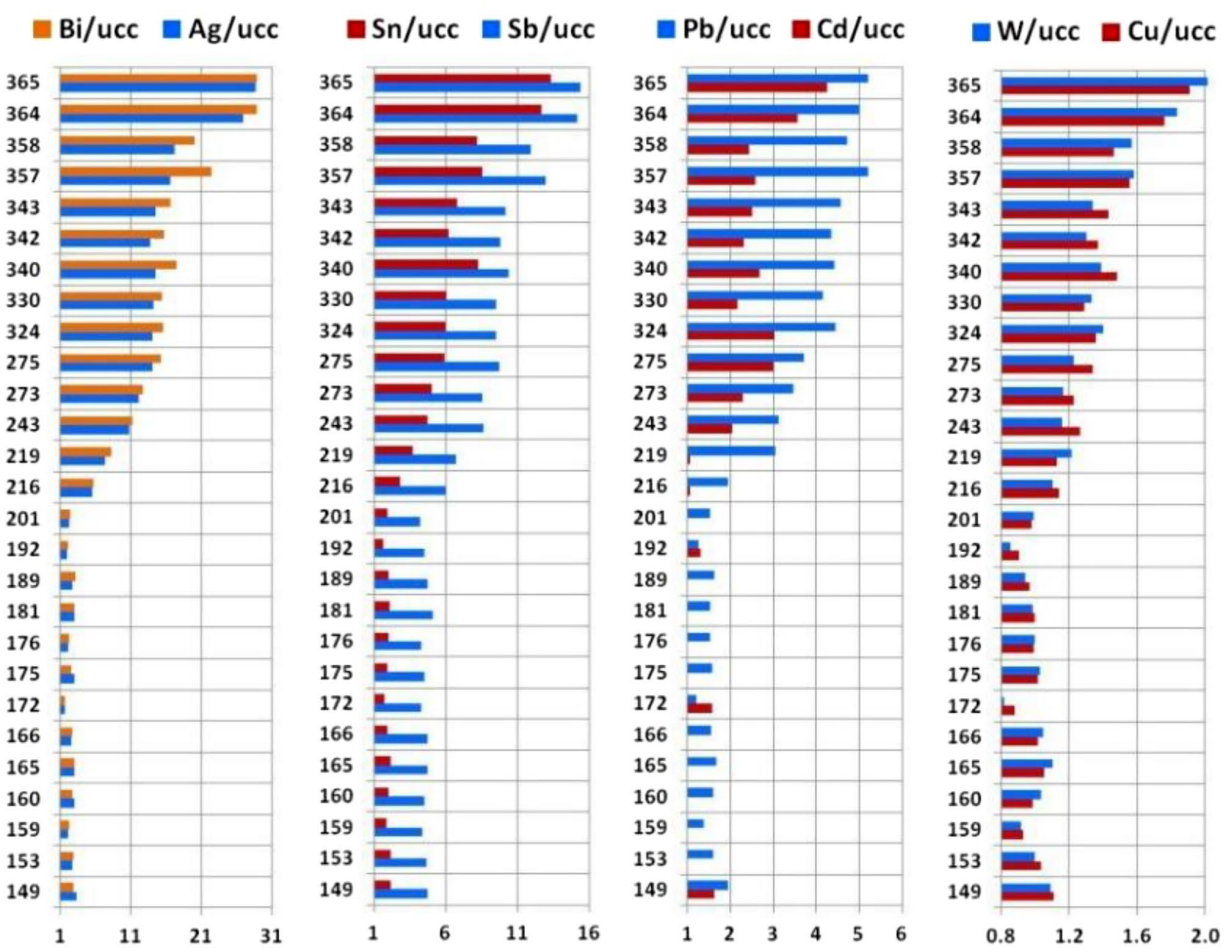
Distribution of chalcophiles and W concentrations in cryoconite of Nalli Glacier sampled at altitudes of 149–365 m a.s.l. The vertical axis shows the absolute altitude of the sampling spot, and the horizontal axis is the normalized contents of the elements. Normalization standard: UCC—Upper Continental Crust^48^.

A summary table of the results of all analytical measurements is attached in Supplementary Table S4. The distribution of concentration variability of a large number of trace elements, as well as several major (macro-) elements, showed clear patterns. The main reference for the normalization of most trace elements was chosen as their generalized average concentrations (Clarke value) in the upper continental crust (UCC)^48^. Macroelements and their ratios are usually reported in weight percentages without normalization. By plotting the distribution of the values obtained for the elements with contrasting behaviour as a function of the altitude of the samples, it is possible to confidently distinguish the two groups. The first group consists of lithophile elements with higher contents in the lower part of the glacier (Fig. 8), which corresponds to the zone A in Figs. 2 and 11. The second group comprises chalcophile elements and W concentrated in the upper part of the sampled area (Fig. 9), i.e., in zone B in Figs. 2 and 11. Similar to radionuclides, we observe a clear distinction between the two altitudinal zones of the glacier at altitudes of 200–220 m a.s.l. (termed an “inversion band”). The established altitude-dependent distribution of concentrations of elements in cryoconite follows general geochemical patterns^49^. The rare earth elements (REEs), Li, Rb, Be, Sc, U, and Th (Fig. 8) are geochemically inert lithophiles. The chalcophiles Bi, Ag, Sn, Sb, Pb, Cd, and Cu (Fig. 9), in turn, are chemically active and mobile under certain geological and geochemical conditions. Accordingly, they are much more affected by human perturbations than lithophiles. Tungsten, which possesses mixed siderophile and lithophile properties, shows a clear relationship with Cu (Fig. 9).

**Figure 10:**
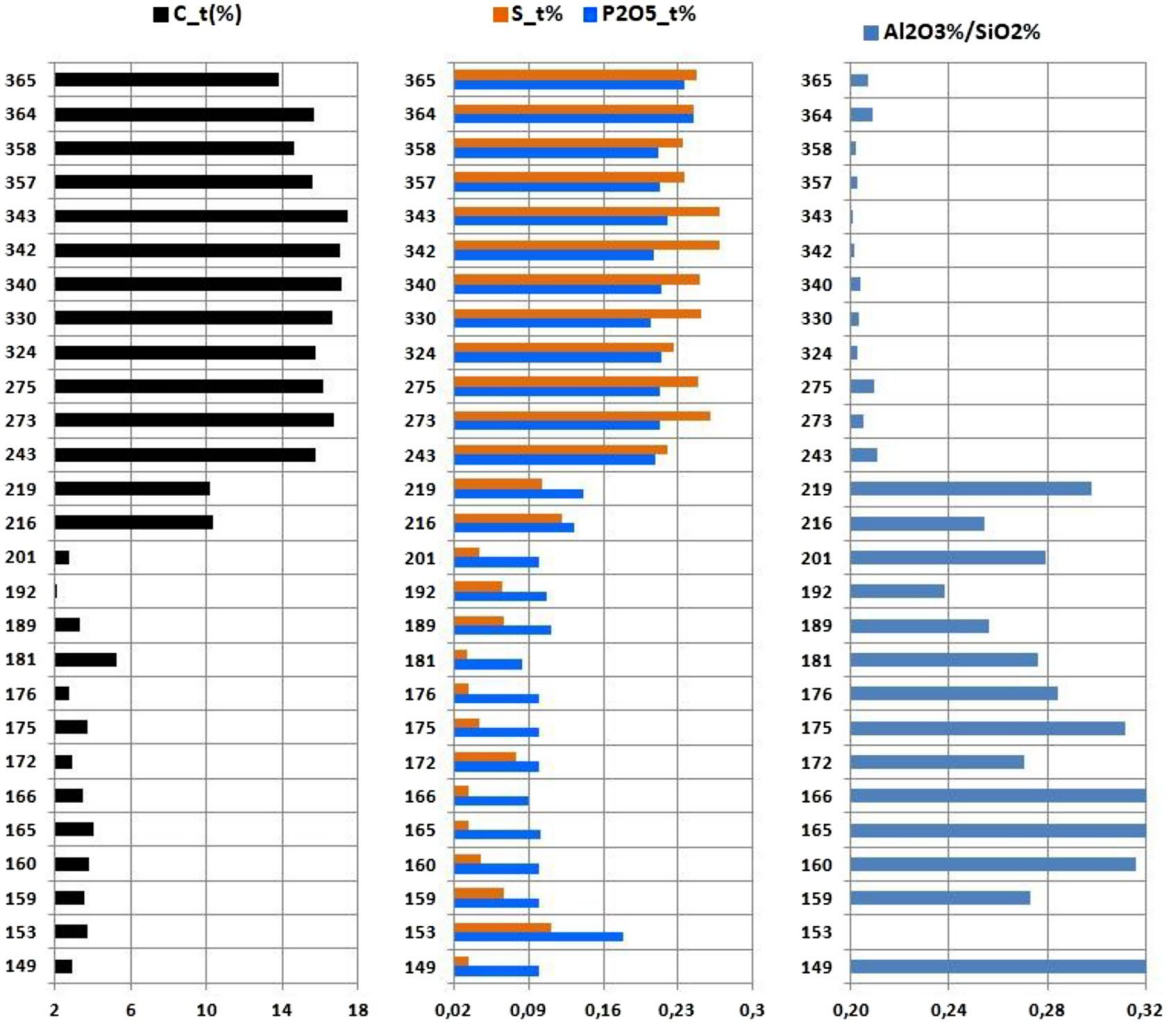
Distribution of C_tot_, S, and P_2_O_5_ contents and Al_2_O_3_/SiO_2_ ratios in cryoconite. The vertical axis shows the absolute altitude of the sampling spot, and the horizontal axis shows the content of chemical components in weight %.

**Figure 11:**
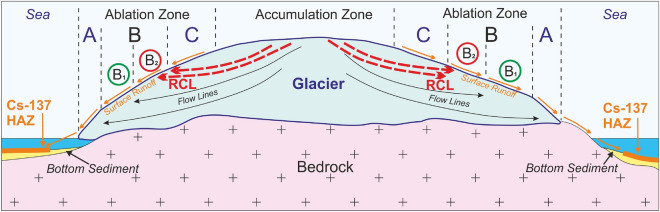
Hypothetic conceptual model of the origin of the zonal distribution of cryoconite composition on the surface of the Novaya Zemlya ice cap. RCL—radioactivity-contaminated layer; A—lower part of the ablation zone in which ^137^Cs activity in cryoconite is below the global fallout level of 58–440 Bq kg^−1^; B—middle part of the ablation zone comprising the following zones: B_1_—zone with ^137^Cs activity at the global fallout level (2700–4700 Bq kg^−1^), B_2_—zone with record-high ^137^Cs activity in cryoconite (5700–8100 Bq kg^−1^); Cs-137 HAZ—high activity zones of radiocesium in bottom sediments. This figure was created using Corel Draw X7 software (https://www.coreldraw.com).

Concentrations of chalcophiles and W in the lower part of the glacier (below 210–220 m a.s.l.) have normalized values close to unity, i.e., are at the level of UCC clarkes. Above the "inversion band", their concentrations gradually increase, and above approximately 350 m a.s.l., another step is visible (Fig. 9). The largest effect is observed for Bi and Ag, showing a 30-fold enrichment with respect to UCC; Sn and Sb show a 15-fold increase. This anomalous jump is observed within zone B above the boundary separating zones B_1_ and B_2_ (Figs. 2 and 11) at the same sampling points where maximum activities of anthropogenic radionuclides are established. The concentrations of some macroelements (C, S, P, Al, and Si) and trace elements are distinctly different in the lower and upper parts of the studied section of the glacier (Fig. 10).

Geochemical analysis reveals the following trends. In the lower part of the glacier (zone A), the contents of U, Th, Be, Sc, Bi, Ag, Pb, Cd, W, Cu, and every individual REE (in Fig. 8, they are grouped into light and heavy REEs: LREEs and HREEs) are close to the UCC Clarke values (see Supplementary Table S4). Only Li, Rb and Sc are slightly enriched. In the upper zone of the glacier (zone B), the contents of REEs, Be, U, and Th are somewhat lower than their clarkes; Li, Rb and Sb are slightly below the reference. However, in the zone B, the concentrations of Pb, Cd, W, Cu, and, in particular, of Bi, Ag, Sn, Sb, are markedly higher than the reference. Concentration of the mentioned elements reached maximum values in the topmost analysed glacier zone (B_2_). These elements often form polymetallic ores, but the nearest large-scale mining and processing plants are 1100 and 1300 km away (Norilsk and Pechenga, respectively). More likely, polymetallic ores of Novaya Zemlya^51^ are the source of these elements. In particular, in 2001 one of the largest Zn–Pb–Ag-containing deposits in Russia was discovered just ~ 80 km south of the Sukhoy Nos test ground (A in Fig. 1)^52^. High exposure (> 95%) of corresponding rocks and numerous outcrops likely promoted entrapment of these elements into explosion clouds, and their subsequent precipitation with radionuclides. This feature of the geological structure of the area explains the extremely high enrichment of surface waters in elements such as Zn, Pb, Sr, Ni, As, Cr, Co, Se, Te, Cd, W, Cu, Sb, and Sn; for many of them, the excess reaches 10-fold with respect to the Clrake values^51^. This hypothesis is supported by obvious correlations between the concentrations of Bi, Ag, Sn, Sb, Pb, Cd, W, and Cu and the activity of anthropogenic radionuclides ^137^Cs, ^241^Am and ^207^Bi. This relationship is obviously related to the simultaneous release of elements and radionuclides from the contaminated ice layer and their entrapment in cryoconite holes.

## Conclusion

Our research reveals the gradual emergence of a new source of radioactive contamination in high latitudes of the Arctic that is essentially secondary by origin. This source is the ice cap of the Severny Island of Novaya Zemlya Archipelago, Russia. Anthropogenic radionuclides (^137^Cs, ^241^Am, and ^207^Bi) produced in atmospheric nuclear tests in the 1950s–1960s were deposited by glaciers. At present, they are released from the ice cap and are fixed on the glacier ablation zone by cryoconite holes at altitudes above 350–370 m a.s.l. The distance to this boundary from the lower termination of the glacier is 4.3 km, and it is approximately 9 km from the glacier accumulation boundary (firn line). The activity of anthropogenic radionuclides, ^210^Pb and the chemical composition of cryoconite located below 210–220 m a.s.l. differ significantly from the one located above. Cryoconite in the upper part of the glacier is several times richer in organic matter, which increases the efficiency of accumulation of both radioactive and selected stable elements. The upper part of the glacier has a shallower angle of surface inclination (1.4°–1.5°), and the impact of meltwater on the stability of cryoconite holes is less pronounced than in the lower part. We believe that the study of cryoconite holes in the topmost part of the Nalli Glacier up to the glacier accumulation boundary (firn line) will allow us to constrain the processes and rates determining the release of elements and isotopes trapped in the radiation-contaminated layer.

## Methods

### Investigation area

Approximately 60% of the Novaya Zemlya glaciation area consists of marine-terminating glaciers, which are the most active parts of ice cap. Nalli Glacier belongs to a relatively rare category of land-terminating glaciers. It is located in the northern part of the Severny Island between the Vershinsky and Moschnyy marine-terminating glaciers (9 and 10 in Fig. 1), with its lower end facing south-southeast towards Blagopoluchiya Bay. According to our observations and data from the Catalogue of Russian Glaciers, its lowest point is located at approximately 70 m a.s.l. at the coordinates 75.7457° N and 63.5968° E. The altitude of the highest point of the glacier is 780 m a.s.l., and the position of the firn line is 610 m a.s.l. The glacier has an area of 195 km^2^ and is approximately 28 km long^15^. No dedicated radar studies of internal structure and ice thickness were performed on Nalli Glacier, but for the adjacent Vershynsky Glacier the thickness is at least 200 m^53^. The rate of glacier movement in the immediate vicinity and assessment of changes in their mass balance have been studied at different times and using different methods for Vershynsky, Moschnyy, Rozhdestvensky, Sredny and Roze Glaciers^53–59^. The average velocities vary from 50 m/year (Roze Glacier) to 200 m/year; the maximal value reaches 350–380 m/year (Vershinsky Glacier)^53^.

Nalli Glacier terminates 5 km inland from Blagopoluchiya Bay. Its southern end is bounded by a rocky ridge with elevations reaching 50 m above the lowest point. The glacier is almost free from fissures (Supplementary Fig. S2). Its limits remain stable in recent decades; in the 1952–2015 period its surface decreased by only 0.35 (± 0.33) km^2^ (~ 0.18%)^57,60^. These facts indicate that the glacier is stable for a long time. Weakly pronounced low-angle overthrusts in the lower part are formed due to differential movements of ice layers^61–63^. Faulting-related surface features are observed at altitudes of 210–22 m a.s.l. as small ridges and grooves (Supplementary Fig. S2) and as a small (3°–3.5°) increase in the surface slope in the lower part. Judging from the stability of the glacier area and position, its mass balance is close to equilibrium. The velocities of the horizontal movement of ice layers at different depths remain unknown.

The area bounded by glaciers and the Kara Sea shoreline, including the shores of Blagopoluchiya Bay, is composed of Upper Devonian mudstones and limestones (D_3_*kl*). They are overlain by silicified and marbleized limestones in the upper part, as well as phthanites, mudstones, carbonate breccias and radiolarians of the lower Carboniferous (C_1_*čr*). They are succeeded by mudstones, siltstones, sandstones with interlayers of limestone, silicites and dolomites of the middle to upper Carboniferous (C_2–3_*kr*)^51,64^.

### Sampling strategy

The sampling strategy was defined after analysis of a preliminary set of samples collected in 2017 and the development of a conceptual hypothetical model, as shown in Fig. 11. This model depicts the formation mechanism of zonality in radiocesium distribution on the Novaya Zemlya ice cap. The zoning originates from the peculiarities of migration of precipitated radionuclides from atmospheric nuclear tests in 1957–1962. We suggest that during that period, fallout of radionuclides occurred in the accumulation zone and formed a (then-buried) contaminated ice layer. Sixty years later, this layer reached the ablation zone, leading to the onset of radionuclides release into meltwater. Cryoconite holes serve as efficient traps for ^137^Cs and other contaminants. During cryoconite formation in the ablation zone at the glacier surface, mineral components and contaminants arrive simultaneously from three sources: (1) contemporary aerosol fallout from the atmosphere, (2) substance release from ice layers entering the melting zone, and (3) overlying cryoconite holes with meltwater released by the glacier. Over time, the density of global fallout decreases, and the contribution of radionuclides from the contaminated ice layer will grow.

Zone C, shown in Fig. 11 as the top part of the ablation zone, is believed to be an ice cap surface lying above the outcrop of the contaminated layer. The upper boundary of zone C coincides with the firn line, which is at 610 m a.s.l^15^. The lowermost boundary corresponds to the contaminated layer outcrop. At present, its elevation is unknown. Assuming that cryoconite holes are younger than 60 years, we expect that in zone C, the radioactivity of cryoconite should be due to global fallout only. Zone B comprises two parts: B_1_ and B_2_. In cryoconite holes of the B_2_ region, two sources of ^137^Cs exist: global fallout and the contaminated layer. The lower margin of B_2_ is the limit of the direct influence of the contaminated layer on cryoconite radioactivity. Released radionuclides are transported by meltwater downstream. At present, this line is at an altitude of ~ 350 m a.s.l., but it will likely move over time. The activity of ^137^Cs in the B_1_ zone should correspond to global fallout at relevant latitudes of 75–80° N^28^. This assumption is confirmed by a comparison of data for Nalli Glacier and Svalbard glaciers (Table 1). The lower boundary of zone B_1_ is at ~ 210–220 m a.s.l. Zone A is the lowermost region, where the environment containing cryoconite holes is rather dynamic and is subject to intense meltwater action. Following this model, consecutive sampling of cryoconite from the lower elevations to the firn line show a gradual increase in the activity of ^137^Cs and other anthropogenic radionuclides. The proposed model may not be universally correct. For example, it is of limited validity for sea-terminated glaciers since their bottom parts experience tensile strain and form fissures with corresponding changes in transport by meltwater.

### Sample collection

Samples were collected from the bottoms of cryoconite holes filled with meltwater using sterile 200 ml Janet syringes (Supplementary Fig. S3a), with a silicone hose attached when necessary (Supplementary Fig. S3b). In cases where the sample was taken from a cryoconite hole only partially filled with water, sterile plastic spoons were used (Supplementary Fig. S3b). The material was then placed in sterile 500 ml polypropylene containers. After returning onboard the ship, the containers were stored in the ship laboratory in a freezer at − 18 °C. Samples were transported frozen to the laboratory in Moscow. In the laboratory, each sample was divided into two parts. One part was returned to the freezer for further storage, and the other part was dried to a constant weight at 60 °C.

### Gamma spectrometry

Measurements of the specific radioactivity of selected samples were carried out in the Department of Chemistry at Lomonosov Moscow State University using a γ-spectrometer with a high purity p-type germanium detector (ORTEC, model GEM-C5060P4-B, 24% relative efficiency, 1.8 keV resolution for 1332 keV line). The detector has a 0.5 mm beryllium window, which allows measurements in the low-energy region. To reduce the natural background, 10 cm passive lead shielding was used. The weight of the samples was 20–100 g. The samples were placed on the detector in 50 ml cylindrical jars. The exposure time of each sample was different and ranged from 60,000 c to 300,000 s. The average spectral acquisition time was 110,000 s. In all measurements, a correction was made for a blank. Measurements were taken within four months after sampling. The spectra were processed using SpectraLine software, LSRM, Russia. Detector efficiency calibration was performed by measuring samples of standard reference material IAEA-447 for natural and artificial radionuclides in moss-soil of different masses. For the ^7^Be line 478 keV and for the ^207^Bi line 569.7 keV, the efficiency was determined by interpolation from the efficiency curve. ^226^Ra was determined using the 609.3 keV ^214^Bi line. Nuclear data were taken from the IAEA database https://nds.iaea.org/relnsd/vcharthtml/VChartHTML.html.

### Chemical analysis

Basic data on microelement concentrations were obtained by inductively coupled plasma–mass spectrometry (ICP MS) on Thermo Elemental equipment at the Analytical Certification Test Centre of the Institute for Microelectronics Technology and High-Purity Materials of the Russian Academy of Sciences. Macronutrient data were taken from X-ray fluorescence analysis (XRF), which was performed using a wavelength-dispersive sequential spectrometer PW 2400 (Philips Analytical) at IGEM RAS Laboratory. Gross carbon contents were determined on a Vario EL elemental analyser at Lomonosov Moscow State University.

### Phase composition and selected properties of cryoconite samples

The phase composition of several samples was studied using X-ray diffraction (XRD) and infrared spectroscopy. X-ray diffraction was performed using an Empyrean diffractometer (Panalytical BV) in Bragg–Brentano geometry using Ni-filtered Cu Kα radiation. Samples were gently manually crushed in ethanol and dried on a zero-background Si holder. Infrared spectra were acquired using a SpectrumOne spectrometer (PerkinElmer) equipped with an AutoImage microscope. The samples were measured on a reflective gold mirror, and the resulting spectra represent the superposition of transmission and reflectance signals with a larger contribution of the former. Digital autoradiography with imaging plates (CyclonePlus scanner, Perkin Elmer) was employed to assess the spatial distribution of radionuclides. The measurements were performed on homogeneously distributed powdered samples. The samples were exposed in a dedicated lead box for periods between 7 and 21 days at room temperature. Longer exposures usually do not provide additional information due to both background accumulation and gradual fading of the stored image^65^. The pixel size in the employed Imaging plates was 42 µm, but since emitted radiation may affect several neighbouring pixels, the practical spatial resolution was somewhat lower. The plates are sensitive to all types of ionizing radiation. In principle, it is possible to discriminate various types of particles^66^, but such experiments require extreme care in sample preparation (very thin samples of uniform thicknesses) and/or use of filters, which is impractical due to a dramatic increase in experiment duration. After autoradiography, the spatial distribution of major chemical elements was studied using a micro-X-ray fluorescence spectrometer (XGT7200V, Horiba) using a 10 micron collimator with excitation energies of 30 and 50 keV. The employed setup also allows acquisition of X-ray transmission images (radiography). In addition, XRF spectra from several points of interest were acquired.

## Supplementary Information

Supplementary Information.
