# Supplementary material for: Radioecological and geochemical peculiarities of cryoconite on Novaya Zemlya glaciers
Source: Sci Rep. 2021 Nov 29;11:23103. doi: 10.1038/s41598-021-02601-8 (PMC8630109; doi:10.1038/s41598-021-02601-8)
Supplement: Supplementary file 1 — Supplementary Information. [file 41598_2021_2601_MOESM1_ESM.pdf]

Supplementary Material to "Radioecological and geochemical peculiarities of cryoconite on Novaya Zemlya glaciers"

Alexey Miroshnikov[1*], Mikhail Flint[2], Enver Asadulin[1], Ramiz Aliev[3,4], Andrei Shiryaev[5,1], Arseniy Kudikov[6], and Vladimir Khvostikov[7]

[1] Institute of Geology of Ore Deposits, Petrography, Mineralogy, and Geochemistry RAS, Moscow, 119017, Russia
[2] Shirshov Institute of Oceanology RAS, Moscow, 117997, Russia
[3] National Research Center "Kurchatov Institute", Moscow, 123182, Russia
[4] Chemistry Department, Lomonosov Moscow State University, Leninskie Gory, 1, Moscow, 119991, Russia
[5] Frumkin Institute of Physical chemistry and Electrochemistry RAS, Moscow, 119071, Russia
[6] Institute of Geography RAS, Moscow, 119017, Russia
[7] Institute of Microelectronics Technology Problems and High-Purity Materials RAS, Chernogolovka, Moscow region, 142432, Russia

* alexey-miroshnikov@yandex.ru

Table S1. Coordinates and elevations of sampling points.

Sample	Latitude (°N)	Longitude (°E)	Altitude a.s.l. (m)
1701	75.7493	63.5878	132.8
1702	75.7507	63.5857	149.0
1703	75.7511	63.5858	153.0
1704	75.7518	63.5860	159.9
1705	75.7524	63.5852	165.0
1706	75.7522	63.5850	166.3
1707	75.7534	63.5904	175.1
1708	75.7532	63.5910	176.2
1709	75.7547	63.5897	188.6
1710	75.7583	63.5997	218.9
1711	75.7566	63.6051	200.8
1712	75.7555	63.6083	191.8
1713	75.7529	63.6018	171.7
1714	75.7518	63.5951	159.0
1801	75.7530	63.5764	181.0
1802	75.7571	63.5716	216.3
1803	75.7622	63.5666	242.8
1804	75.7675	63.5523	273.0
1805	75.7677	63.5534	274.9
1806	75.7735	63.5416	324.0
1807	75.7744	63.5406	330.4
1808	75.7766	63.5331	339.5
1809	75.7781	63.5281	342.4
1810	75.7782	63.5266	342.8
1811	75.7835	63.5195	357.2
1812	75.7840	63.5198	358.0
1813	75.7865	63.5307	363.8
1814	75.7871	63.5306	365.3

Table S2. Activity of anthropogenic and natural radionuclides in cryoconite samples from Nalli Glacier (Bq kg^{-1} d.m.).

Sample	^{137}Cs	^{241}Am	^{207}Bi	^{210}Pb (total)	^{226}Ra	^{7}Be
1701	58 (±2)	<LD	< LD	1283 (±31)	34(±3)	44 (±13)
1702	208 (±5)	< LD	< LD	5823 (±155)	31(±4)	1264 (±45)
1703	270 (±4)	1.8 (±0.9)	< LD	2566 (±46)	31(±2)	121 (±20)
1704	275 (±5)	1.8 (±0.9)	< LD	2449 (±42)	37(±3)	1199 (±36)
1705	313 (±5)	< LD	< LD	2951 (±71)	33(±3)	553 (±37)
1706	207 (±2)	1.8 (±0.8)	< LD	2419 (±46)	29(±1)	469 (±16)
1707	270 (±3)	1.6 (±0.9)	< LD	2573 (±41)	33(±2)	82 (±19)
1708	215 (±4)	1.4 (±0.6)	< LD	2261 (±43)	30(±2)	420 (±32)
1709	436 (±5)	2.8 (±0.7)	< LD	2408 (±46)	28(±2)	354 (±20)
1710	2076 (±20)	9.1 (±2.7)	2.1 (±0.7)	5134 (±117)	22(±4)	1143 (±52)
1711	210 (±2)	1.3 (±0.8)	< LD	2300 (±37)	31(±1)	89 (±11)
1712	140 (±2)	1.5 (±0.7)	< LD	1381 (±28)	28(±1)	122 (±11)
1713	75 (±2)	< LD	< LD	1454 (±26)	31(±1)	73 (±9)
1714	203 (±2)	2.1 (±0.9)	< LD	1863 (±39)	33(±1)	38 (±10)
1801	244 (±4)	< LD	< LD	2758 (±46)	32(±2)	94 (±13)
1802	1046 (±15)	4.4 (±1.3)	< LD	6004 (±160)	29(±4)	2418 (±76)
1803	2667 (±5)	12.5 (±0.8)	2.2 (±0.4)	8873 (±212)	20(±2)	1000 (±30)
1804	2991 (±27)	16.5 (±1.1)	2.6 (±0.6)	9748 (±274)	20(±2)	1076 (±53)
1805	3215 (±30)	18.8 (±1.1)	2.9 (±0.7)	9716 (±290)	21(±4)	957 (±43)
1806	4659 (±34)	28.1 (±1.0)	3.8 (±0.5)	9530 (±304)	22(±2)	474 (±78)
1807	3745 (±32)	22.0 (±1.3)	3.0 (±0.7)	6488 (±105)	20(±2)	224 (±46)
1808	4420 (±40)	26.7 (±1.5)	4.4 (±0.8)	6775 (±154)	17(±3)	414 (±48)
1809	3650 (±29)	23.0 (±2.2)	3.8 (±0.8)	7232 (±86)	17(±3)	356 (±53)
1810	3531 (±29)	20.7 (±1.2)	4.4 (±0.7)	8484 (±247)	19(±2)	481 (±46)
1811	5665 (±44)	48.0 (±1.2)	6.3 (±0.6)	6641 (±234)	19(±2)	161 (±51)
1812	5640 (±52)	48.8 (±2.0)	5.4 (±0.9)	9175 (±96)	22(±3)	898 (±70)
1813	8093 (±69)	58.3 (±2.3)	5.3 (±1.2)	6888 (±315)	18(±3)	775 (±107)
1814	7125 (±56)	48.8 (±2.0)	5.1 (±1.1)	8327 (±117)	15(±3)	572 (±92)

Table S3. Activity of anthropogenic and natural radionuclides in cryoconite samples from Jotufonna (J), Vestre Grønfjordbreen (G) and Fridtjovbreen (F) glaciers, Svalbard (Bq kg^{-1} d.m.).

Sample	^{137}Cs	^{241}Am	^{207}Bi	^{210}Pb (total)	^{226}Ra	^{7}Be
J1701	17 (±1)	<LD	<LD	519 (±9)	65 (±2)	30 (±8)
J1702	289 (±3)	2.3 (±0.9)	0.7 (±0.3)	1132 (±29)	64 (±2)	<LD
J1703	531 (±4)	4.7 (±1.1)	0.7 (±0.2)	1176 (±37)	66 (±2)	74 (±12)
J1704	1114 (±10)	8.4 (±1.3)	1.7 (±0.5)	2523 (±40)	58 (±3)	41 (±15)
J1705	344 (±3)	2.8 (±1.0)	0.6 (±0.3)	2163 (±41)	59 (±2)	60 (±7)
G1801	111 (±2)	<LD	<LD	2843 (±57)	51 (±2)	481 (±41)
G1802	106 (±3)	<LD	<LD	3688 (±72)	45 (±2)	341 (±30)
F1801	11 (±1)	<LD	<LD	1485 (±27)	55 (±1)	1720 (±32)
F1802	496 (±5)	5.6 (±0.8)	<LD	1965 (±51)	64 (±2)	499 (±32)
F1803	1781 (±16)	24.0 (±1.4)	2.6 (±0.6)	4718 (±69)	49 (±2)	445 (±45)
F1804	153 (±2)	1.2 (±1.1)	<LD	1935 (±46)	65 (±1)	<LD

Table S4. Composition of the samples determined through XRF and ICP MS. Gross carbon contents were determined on a Vario EL elemental analyser. Please note that the unit is not the same for all the elements.

Sample #		1701	1702	1703	1704	1705	1706	1707	1708	1709	1710	1711	1712	1713	1714	1801	1802	1803	1804	1805	1806	1807	1808	1809	1810	1811	1812	1813	1814
Na_2O%	wt%	0.78	0.84	0.97	0.85	0.84	0.85	0.84	1.05	1.04	0.92	1.04	1.03	1.07	1.05	0.77	0.83	0.96	0.93	0.91	0.92	0.89	0.92	0.88	0.89	0.88	0.88	0.86	0.87
MgO%		2.26	2.25	2.38	2.22	2.24	2.28	2.21	2.21	2.11	2.11	2.17	2.29	2.46	2.29	2.53	2.32	2.16	2.09	2.11	2.09	2.08	2.07	2.01	2.03	2.08	2.10	2.08	2.09
Al_2O_3%		20.49	21.15	14.04	20.28	20.92	21.19	20.05	18.63	17.20	19.32	18.37	16.13	16.81	17.18	17.89	16.68	14.42	14.11	14.36	14.01	14.05	14.04	13.91	13.88	14.03	13.99	14.37	14.23
K_2O%		3.29	3.51	2.82	3.44	3.62	3.69	3.43	3.47	3.19	3.33	3.32	2.78	3.04	3.16	3.39	3.08	2.67	2.66	2.67	2.64	2.67	2.67	2.70	2.71	2.69	2.65	2.67	2.69
CaO%		0.48	0.23	0.65	0.24	0.19	0.19	0.21	0.18	0.28	0.46	0.39	1.42	6.55	5.23	0.32	0.48	0.74	0.71	0.71	0.70	0.69	0.69	0.67	0.67	0.65	0.63	0.65	0.64
TiO_2%		1.20	1.19	0.94	1.14	1.22	1.20	1.14	1.05	1.01	1.27	1.07	0.93	0.92	0.98	1.13	1.26	1.26	1.26	1.24	1.24	1.26	1.25	1.25	1.26	1.24	1.22	1.24	1.23
Fe_2O_3%*		8.08	7.72	5.50	7.52	7.53	7.53	7.61	7.75	7.81	7.48	7.74	7.54	6.80	7.06	9.07	9.58	8.85	8.85	8.93	8.72	8.66	8.95	8.92	8.91	8.60	8.69	8.82	8.95
P_2O_5%*		0.11	0.10	0.18	0.10	0.10	0.09	0.10	0.10	0.11	0.14	0.10	0.11	0.10	0.10	0.08	0.13	0.21	0.21	0.21	0.21	0.20	0.21	0.21	0.22	0.21	0.21	0.24	0.24
S%*		0.05	0.03	0.11	0.04	0.03	0.03	0.04	0.03	0.07	0.10	0.04	0.06	0.08	0.07	0.03	0.12	0.22	0.26	0.25	0.23	0.25	0.25	0.27	0.27	0.24	0.23	0.24	0.25
C%*		2.07	2.87	3.71	3.78	4.02	3.42	3.73	2.77	3.30	10.16	2.71	2.07	2.87	3.56	5.25	10.34	15.75	16.72	16.14	15.78	16.66	17.14	17.04	17.44	15.61	14.64	15.70	13.83
Be	mg/kg	2.31	2.45	2.54	2.37	2.39	2.41	2.32	2.47	2.37	1.85	2.35	2.12	2.05	2.07	2.41	1.85	1.31	1.26	1.32	1.29	1.17	1.36	1.26	1.32	1.38	1.32	1.42	1.44
Sc		18.2	15.7	18.1	20.1	20.4	20.5	18.8	19.3	16.6	17.7	19.0	17.4	14.9	16.7	18.6	17.5	14.2	12.9	13.4	13.6	12.8	13.1	13.0	13.1	13.3	13.6	12.9	13.6
V		136.4	138.2	140.6	115.8	135.6	121.4	118.1	145.5	130.5	125.9	134.4	120.4	122.2	123.2	138.3	132.1	109.8	91.7	99.2	102.9	92.7	102.8	95.2	99.0	100.1	79.1	96.0	100.6
Co		21.9	17.7	16.4	16.5	16.5	16.8	17.0	16.9	16.3	13.3	16.4	17.6	16.9	16.3	15.9	14.2	10.9	9.9	10.6	10.1	9.2	10.2	9.8	10.0	10.2	10.1	10.5	11.1
Cu		30.7	31.0	28.9	27.5	29.5	28.4	28.4	27.7	27.1	31.5	27.4	25.3	24.7	25.9	28.0	32.0	35.4	34.4	37.5	38.0	36.1	41.4	38.4	40.0	43.6	40.9	49.3	53.5
Ga		22.4	23.1	23.5	22.5	23.0	24.0	22.5	23.2	21.9	20.5	22.0	20.3	18.7	20.3	22.8	19.7	16.9	16.0	17.6	17.6	16.7	18.1	16.8	17.4	19.6	18.9	20.1	20.5
Se		2.51	2.79	2.78	2.98	2.70	2.69	2.55	2.69	2.72	3.64	2.57	2.45	2.36	2.41	3.12	3.27	5.23	5.03	4.73	4.32	4.20	3.90	4.31	4.29	3.79	3.50	3.79	4.44
Rb		142	152	151	149	156	163	145	151	139	121	149	124	128	138	151	117	80.9	75.1	78.2	80.4	75.3	78.6	76.6	79.2	82.1	82.8	79.2	81.4
Sr		175	177	177	171	179	189	170	181	151	141	179	147	226	242	179	155	96.4	88.2	93.5	92.5	86.4	90.7	86.5	88.0	94.1	91.5	91.5	93.4
Y		39.6	34.3	35.2	36.6	34.8	34.3	36.9	35.9	36.5	30.4	35.1	38.3	32.1	32.8	32.7	33.7	23.7	21.8	22.8	22.1	19.9	20.5	20.2	20.3	21.2	22.0	21.4	21.4
Zr		225	198	183	179	175	183	186	181	186	184	185	188	160	166	176	195	169	144	156	146	136	142	138	145	150	150	146	151
Nb		17.3	17.0	16.4	16.2	17.1	17.3	16.4	16.4	15.3	15.9	15.9	14.5	13.1	14.4	15.8	15.5	13.3	12.7	12.9	12.9	12.1	12.9	12.4	12.5	13.2	13.0	12.7	13.4
Mo		2.00	2.14	2.01	1.82	2.20	2.08	1.92	1.80	2.17	2.48	1.79	1.71	2.62	1.66	2.20	2.66	3.01	2.80	3.02	3.47	2.74	3.24	2.81	4.94	3.06	2.72	2.99	3.12
Ag		0.15	0.18	0.14	0.16	0.16	0.14	0.16	0.11	0.14	0.39	0.12	0.11	0.09	0.11	0.16	0.29	0.57	0.64	0.74	0.75	0.75	0.77	0.73	0.76	0.88	0.91	1.4	1.5
Cd		0.13	0.15	0.08	0.07	0.06	0.06	0.07	0.06	0.09	0.10	0.05	0.12	0.14	0.04	<0.04	0.10	0.18	0.21	0.27	0.27	0.20	0.24	0.21	0.23	0.23	0.22	0.32	0.38
Sn		3.9	4.5	4.5	4.2	4.5	4.1	4.1	4.2	4.3	7.8	4.1	3.4	3.6	3.9	4.3	5.9	9.9	10.5	12.4	12.6	12.7	17.3	13.0	14.3	18.0	17.3	26.6	28.1
Sb		1.91	1.91	1.86	1.80	1.89	1.89	1.81	1.72	1.90	2.68	1.70	1.81	1.73	1.75	2.04	2.38	3.44	3.43	3.90	3.80	3.80	4.17	3.94	4.08	5.19	4.79	6.06	6.16
Cs		6.93	7.46	7.59	7.40	7.75	7.99	7.28	7.66	6.79	6.82	7.26	5.87	6.01	6.71	7.49	6.43	5.28	4.90	5.21	5.37	5.18	5.44	5.26	5.33	5.72	5.56	5.63	5.80
Ba		425	457	460	453	471	479	455	468	431	421	442	371	389	402	474	404	344	320	337	339	322	340	326	332	350	345	341	350
La		45.9	36.4	35.3	36.4	36.9	35.0	36.4	36.3	36.7	35.4	35.4	37.6	34.1	33.9	33.5	34.3	29.7	25.6	27.4	27.1	25.6	25.6	25.6	25.7	26.2	27.3	25.3	28.2
Ce		96.0	75.8	73.1	75.1	76.9	72.7	75.5	75.5	75.9	72.6	73.2	78.5	71.3	70.7	69.6	70.5	61.8	52.2	57.3	56.0	53.4	53.7	52.8	53.5	55.9	56.8	53.2	57.6
Pr		11.4	9.3	8.8	9.0	9.1	8.7	9.0	9.0	9.1	8.4	8.8	9.4	8.6	8.4	8.2	8.3	7.1	5.9	6.5	6.4	6.0	6.0	6.0	6.0	6.3	6.3	6.0	6.5
Nd		44.9	36.2	34.4	35.7	36.1	34.2	35.7	35.5	35.4	33.3	34.5	36.8	34.3	33.2	32.1	32.7	27.7	23.1	25.2	24.7	23.3	22.8	23.2	23.1	24.1	24.7	22.7	24.7
Sm		8.86	7.16	6.93	6.95	7.02	6.81	7.02	6.97	7.03	6.29	6.78	7.40	6.88	6.61	6.35	6.27	5.26	4.34	4.78	4.58	4.32	4.40	4.37	4.33	4.72	4.65	4.42	4.70
Eu		1.71	1.36	1.32	1.37	1.36	1.32	1.36	1.34	1.38	1.25	1.32	1.46	1.34	1.28	1.23	1.25	1.06	0.91	0.97	0.94	0.87	0.92	0.91	0.88	0.96	0.94	0.89	0.94
Gd		7.18	5.76	5.59	5.87	5.69	5.50	5.85	5.89	5.88	5.05	5.68	6.17	5.70	5.48	5.24	5.13	4.10	3.56	3.74	3.65	3.40	3.40	3.37	3.47	3.58	3.71	3.33	3.63
Tb		1.17	0.97	0.95	0.99	0.96	0.92	1.00	1.00	1.00	0.84	0.96	1.04	0.94	0.91	0.89	0.88	0.66	0.59	0.63	0.60	0.55	0.54	0.57	0.57	0.58	0.61	0.57	0.60
Dy		7.22	6.31	5.93	6.51	6.25	5.69	6.46	6.28	6.14	5.37	6.30	6.53	5.89	5.81	5.38	5.70	4.19	3.72	3.70	3.88	3.51	3.40	3.60	3.63	3.53	3.85	3.46	3.77
Ho		1.46	1.30	1.27	1.31	1.24	1.21	1.32	1.28	1.30	1.10	1.29	1.39	1.18	1.19	1.14	1.18	0.83	0.77	0.82	0.80	0.71	0.73	0.73	0.74	0.76	0.77	0.75	0.75
Er		4.51	3.98	3.88	4.11	3.91	3.81	4.17	3.98	4.03	3.40	3.98	4.20	3.71	3.67	3.59	3.68	2.63	2.41	2.51	2.46	2.22	2.30	2.29	2.30	2.43	2.40	2.38	2.36
Tm		0.63	0.55	0.52	0.56	0.55	0.53	0.57	0.55	0.54	0.47	0.54	0.56	0.50	0.51	0.47	0.52	0.36	0.33	0.34	0.34	0.31	0.31	0.32	0.33	0.33	0.34	0.32	0.34
Yb		4.25	3.79	3.61	3.68	3.64	3.50	3.79	3.76	3.77	3.19	3.59	3.87	3.34	3.48	3.36	3.41	2.43	2.26	2.33	2.30	2.13	2.14	2.13	2.20	2.27	2.32	2.19	2.23
Lu		0.61	0.55	0.51	0.55	0.53	0.51	0.54	0.55	0.53	0.46	0.54	0.55	0.50	0.51	0.49	0.49	0.36	0.33	0.36	0.34	0.32	0.32	0.32	0.31	0.34	0.33	0.33	0.34
Hf		6.05	5.37	4.81	4.97	4.91	4.83	5.04	4.84	4.99	4.93	5.04	5.02	4.35	4.62	4.61	5.19	4.44	3.80	4.27	3.96	3.62	3.78	3.68	3.87	4.41	3.93	4.01	4.00
Ta		1.25	1.22	1.15	1.20	1.24	1.22	1.16	1.17	1.07	1.14	1.15	1.01	0.97	1.03	1.11	1.13	0.95	0.89	0.90	0.94	0.88	0.93	0.88	0.90	0.95	0.93	0.92	0.99
W		2.00	2.07	1.89	1.97	2.10	1.99	1.95	1.89	1.79	2.31	1.88	1.63	1.56	1.74	1.87	2.10	2.20	2.21	2.32	2.66	2.53	2.63	2.48	2.54	3.01	2.98	3.48	3.84
Tl		0.90	0.96	0.96	0.93	1.00	1.02	0.92	0.98	0.86	0.79	0.93	0.77	0.79	0.85	0.94	0.75	0.56	0.53	0.55	0.59	0.55	0.59	0.58	0.58	0.64	0.62	0.63	0.67
Pb		24.0	32.8	27.3	27.1	28.4	26.5	26.7	25.9	27.4	51.8	25.9	21.4	20.6	23.4	25.7	32.8	53.1	58.9	62.9	75.7	70.8	75.3	74.1	77.8	88.6	80.0	85.3	88.5
Bi		0.34	0.46	0.45	0.44	0.49	0.42	0.41	0.37	0.50	1.32	0.39	0.33	0.27	0.35	0.47	0.90	1.79	2.03	2.43	2.49	2.46	2.78	2.52	2.64	3.57	3.21	4.61	4.60
Th		12.9	10.4	10.9	12.0	12.3	11.7	11.2	11.4	10.6	10.8	11.3	11.1	9.6	10.1	10.7	10.8	8.8	8.0	8.6	8.9	8.2	8.3	8.3	8.3	8.6	8.5	8.4	8.9
U		3.28	3.10	2.88	2.94	3.02	2.92	2.87	2.90	2.82	2.85	2.82	2.77	2.70	2.71	2.95	3.17	2.37	2.15	2.28	2.32	2.16	2.22	2.13	2.16	2.32	2.23	2.24	2.32

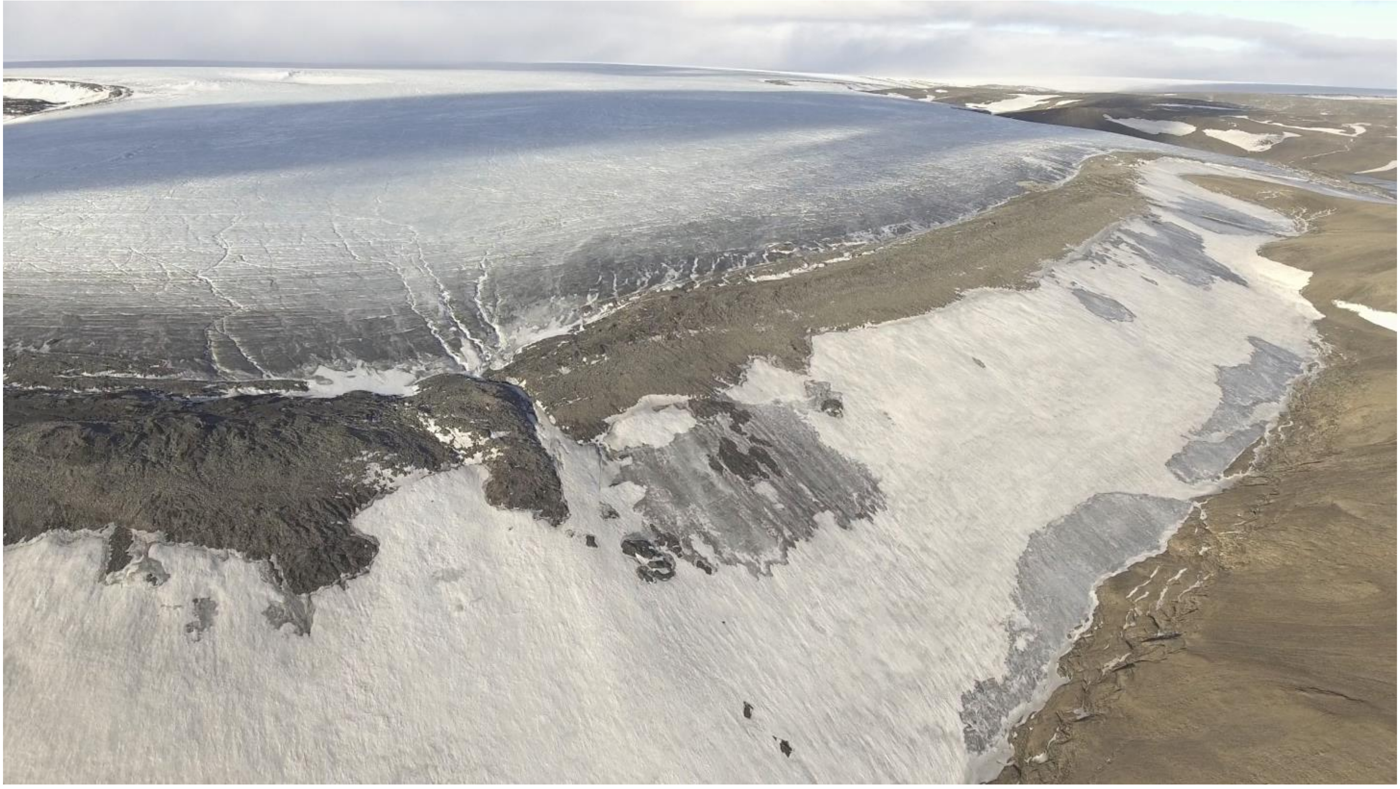

Figure S1. Nalli Glacier as seen from ~170 m elevation. Photo by Evgeniy Bogatov with permission under a CC BY open access license.

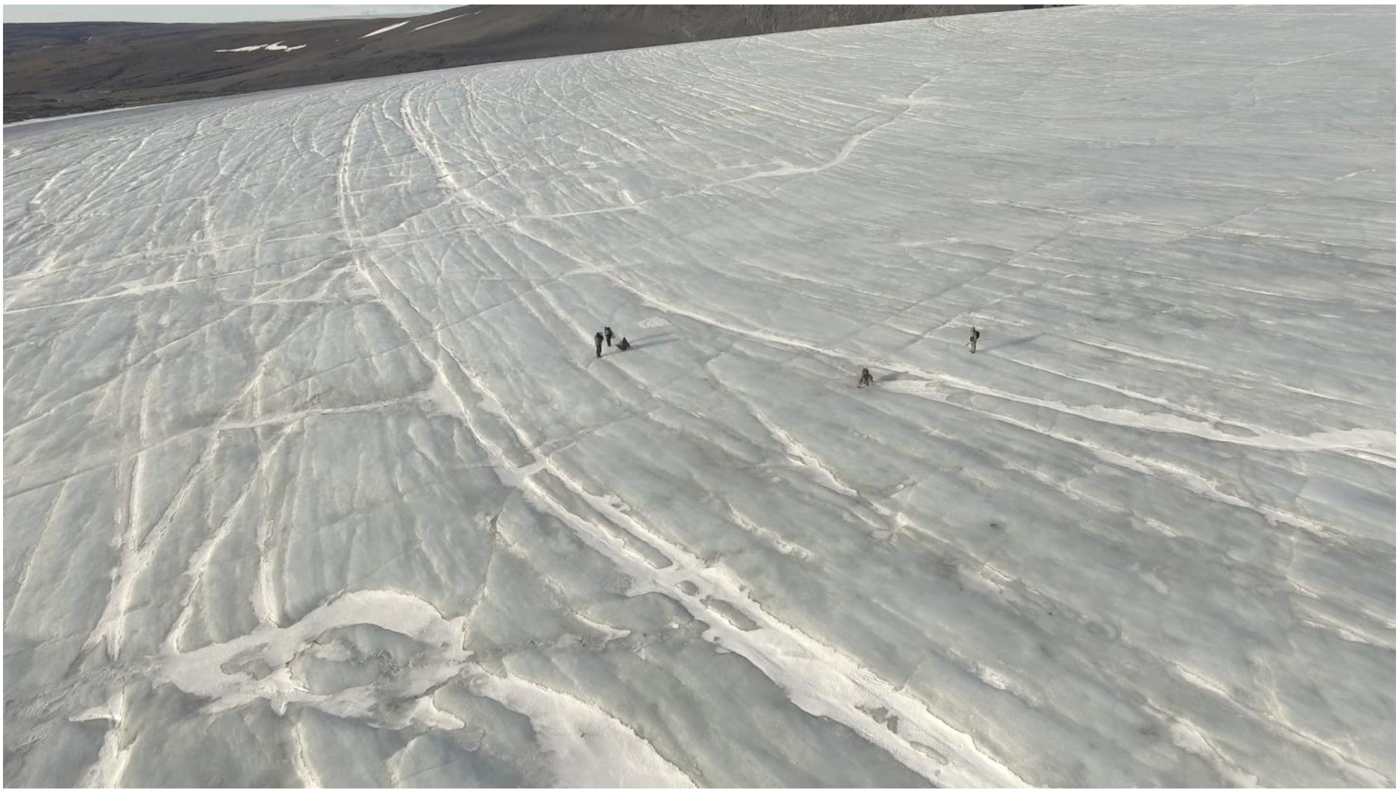

Figure S2. Nalli Glacier as seen from ~100 m elevation. Photo by Evgeniy Bogatov with permission under a CC BY open access license.

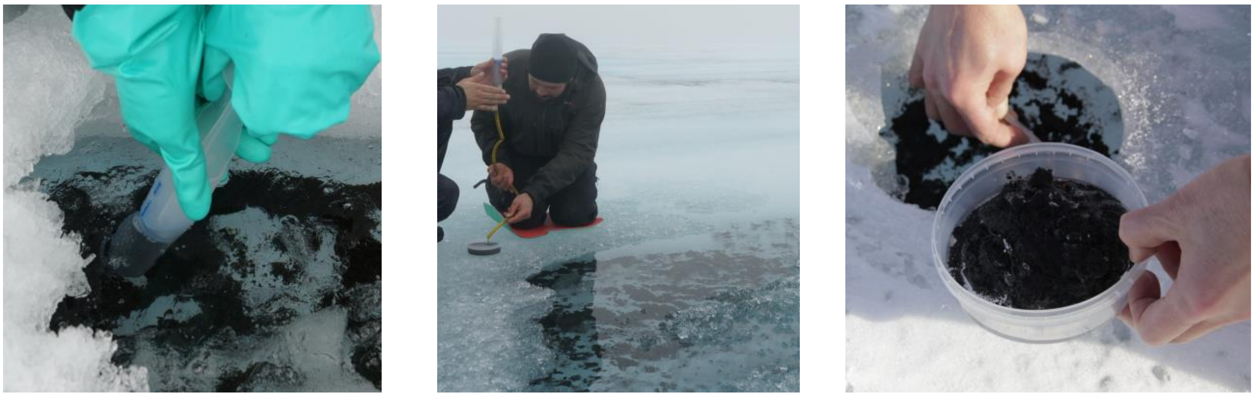

a b c

Figure S3. Collection of sediments from cryoconite holes using Janet's syringe (a and b) or a plastic spoon (c). Photo by Evgeniy Bogatov with permission under a CC BY open access license.
